# Supplementary material for: Probiotics, prebiotics and synbiotics for chronic kidney disease: protocol for a systematic review and meta-analysis
Source: BMJ Open. 2018 Jul 28;8(7):e020863. doi: 10.1136/bmjopen-2017-020863 (PMC6067341; doi:10.1136/bmjopen-2017-020863)
Supplement: Supplementary file 1 [file bmjopen-2017-020863supp001.pdf]

Appendix 1.

The search strategy for the Pubmed database:

1. Kidney Diseases/
2. Renal Insufficiency/
3. exp Renal Insufficiency, Chronic/
4. (kidney disease* or renal disease* or kidney failure or renal failure or kidney insufficienc* or renal insufficienc* or frasier syndrome).tw.
5. (ESRF or ESKF or ESRD or ESKD).tw.
6. (CKF or CKD or CRF or CRD).tw.
7. or/1-6
8. Probiotics/
9. probiotic*.tw.
10. Synbiotics/
11. synbiotic*.tw.
12. Prebiotics/
13. prebiotic*.tw.
14. exp Lactobacillus/
15. Lactobacillus.tw.
16. lactobacilli*.tw.
17. exp Bifidobacterium/
18. bifidobacteri*.tw.
19. exp Bacillus/
20. bacilli*.tw.
21. bacillu*.tw.
22. Clostridium butyricum/
23. clostridium butyricum.tw.
24. Streptococcus thermophilus/
25. streptococcus thermophill*.tw.
26. exp Escherichia coli/
27. (escherichia coli or E coli or EAggEC).tw.

28. exp Propionibacterium/
29. propionibacteria*.tw.
30. propionibacterium freudenreich*.tw.
31. exp Enterococcus faecium/
32. enterococcus faecium.tw.
33. streptococcus faecium.tw.
34. exp Saccharomyces/
35. (saccharomyces boulardi or Saccharomyces cerevisiae or Baker Yeast* or S cerevisiae or Bakers Yeast or Brewer* Yeast).tw.
36. vsl3.tw.
37. vsl 3.tw.
38. ganedenBc.tw.
39. lafti b94.tw.
40. mutaflor.tw.
41. actimel.tw.
42. cultura.tw.
43. yakult.tw.
44. l casei.tw.
45. shirota.tw.
46. Enterobacteriaceae/
47. proviva.tw.
48. vifit.tw.
49. verum.tw.
50. bio K.tw.
51. multibionta.tw.
52. oligofructose.mp
53. Inulin/
54. Inulin.tw
55. or/8-54
56. 7 and 55

57. randomized controlled trial.pt.
58. controlled clinical trial.pt.
59. randomised.tw.
60. randomized.tw.
61. placebo.tw.
62. randomly.tw.
63. trial.tw.
64. groups.tw.
65. or/57-64
66. exp animals/ not humans.sh.
67. 65 not 66
68. 56 and 67
