# Supplementary material for: Probiotics, prebiotics and synbiotics for chronic kidney disease: protocol for a systematic review and meta-analysis
Source: BMJ Open. 2018 Jul 28;8(7):e020863. doi: 10.1136/bmjopen-2017-020863 (PMC6067341; doi:10.1136/bmjopen-2017-020863)
Supplement: Author's manuscript [file bmjopen-2017-020863.draft_revisions.pdf]

BMJ Open

BMJ Open is committed to open peer review. As part of this commitment we make the peer review history of every article we publish publicly available.

When an article is published we post the peer reviewers' comments and the authors' responses online. We also post the versions of the paper that were used during peer review. These are the versions that the peer review comments apply to.

The versions of the paper that follow are the versions that were submitted during the peer review process. They are not the versions of record or the final published versions. They should not be cited or distributed as the published version of this manuscript.

BMJ Open is an open access journal and the full, final, typeset and author-corrected version of record of the manuscript is available on our site with no access controls, subscription charges or pay-per-view fees (http://bmjopen.bmj.com).

If you have any questions on BMJ Open's open peer review process please email info.bmjopen@bmj.com

Probiotics, prebiotics and synbiotics for chronic kidney disease: protocol for a systematic review and meta-analysis

Journal:	*BMJ Open*
Manuscript ID	bmjopen-2017-020863
Article Type:	Protocol
Date Submitted by the Author:	28-Nov-2017

Complete List of Authors:	Pei, Ming; First Teaching Hospital of Tianjin University of Traditional Chinese Medicine, Division of Nephrology<br>Wei, Lijuan; Tianjin University of Traditional Chinese Medicine, Graduate College<br>Hu, Shouci; Tianjin University of Traditional Chinese Medicine, Graduate College<br>Yang, Bo; First Teaching Hospital of Tianjin University of Traditional Chinese Medicine, Division of Nephrology<br>Si, Jinhua; Tianjin University of Traditional Chinese Medicine, Library<br>Yang, Hongtao; First Teaching Hospital of Tianjin University of Traditional Chinese Medicine, Division of Nephrology<br>Zhai, Jingbo; Tianjin University of Traditional Chinese Medicine, Research institute of Traditional Chinese Medicine

Keywords:	Probiotics, prebiotics, synbiotics, chronic kidney disease, systematic review, meta-analysis

SCHOLARONE™ Manuscripts

For peer review only - http://bmjopen.bmj.com/site/about/guidelines.xhtml

Probiotics, prebiotics and synbiotics for chronic kidney disease: protocol for a systematic review and meta-analysis

Ming Pei[1], Lijuan Wei[2], Shouci Hu[2], Bo Yang[1], Jinhua Si[3], Hongtao Yang[1*], Jingbo Zhai[4*]

[1] Division of Nephrology, First Teaching Hospital of Tianjin University of Traditional Chinese Medicine,314 Anshanxi Road, Nankai District, Tianjin 300193, China;

[2] Graduate College, Tianjin University of Traditional Chinese Medicine, 312 Anshanxi Road,Nankai District, Tianjin 300193, China;

[3]Library of Tianjin University of Traditional Chinese Medicine, 312 Anshanxi Road, Nankai District, Tianjin 300193, China;

[4] Research institute of Traditional Chinese Medicine, Tianjin University of Traditional Chinese Medicine, 312 Anshanxi Road,Nankai District, Tianjin 300193, China;

Ming Pei and Lijuan Wei contributed equally.

Correspondence to Hongtao Yang, tjtcmht@126.com; Jingbo Zhai, zhaijingbo@foxmail.com

ABSTRACT

Introduction:

There is growing interest in probiotic, prebiotic and synbiotic supplements for patients with chronic kidney disease (CKD). However, no comprehensive systematic review has been conducted to evaluate the efficacy and safety of probiotics, prebiotics or sybiotics for CKD patients without dialysis and renal transplantation. We aim to assess the efficacy and safety of probiotics, prebiotics and sybiotics for non-dialysis or non-renal transplant patients with CKD.

Methods and analysis:

An extensive literature search will be undertaken to identify potentially eligible studies in the electronic databases PubMed, EMBASE, Web of Science, and Cochrane Central Register of Controlled Trials (CENTRAL). No language restriction will be applied to the search. Parallel-group and crossover randomised controlled trials will be included. The 'risk of bias' of each included study will be assessed using the Cochrane 'risk of bias' tool. The primary outcome is uraemic toxins (including but not limited to indoxyl sulphate and p-cresyl sulphate). The secondary outcomes include kidney function, adverse cardiovascular events, all-cause mortality and cause-specific death, progression to ESKD, quality of life, and adverse events. The data will be synthesised with appropriate statistical methods. The quality of evidence for each

For peer review only - http://bmjopen.bmj.com/site/about/guidelines.xhtml

outcome will be assessed using the GRADE approach.

Ethics and dissemination:

No primary data will be collected, so ethical approval is not required. We will publish findings from this systematic review in a peer-reviewed scientific journal, and the data set will be made freely available.

This protocol has been registered on PROSPERO (CRD42017079177).

Strengths and limitations of this study

- This study will assess the efficacy and safety of probiotics, prebiotics or sybiotics for non-dialysis or non-renal transplant patients with chronic kidney disease (CKD).
- This protocol was developed following the Preferred Reporting Items for Systematic reviews and Meta-Analyses guidelines for protocols (PRISMA-P).
- It will be fundamental for reliable recommendations in the management of CKD.
- Study heterogeneity will likely pose challenges for the meta-analysis.

BACKGROUND

Chronic kidney disease (CKD) is one of the most common chronic diseases worldwide[1]. It was defined as abnormalities of kidney structure or function, present for more than 3 months, with implications for health by Kidney Disease: Improving Global Outcomes (KDIGO)[2]. KDIGO categorised the severity of CKD into five stages according to glomerular filtration rate (GFR) and three stages based on albuminuria[3].

The incidence of CKD is subject to large variation worldwide[4]. The prevalence of CKD is globally estimated to be 8-16%, with 10.8% in China and 13.1% in America[5,6]. Diabetes and hypertension are the main risk factors for CKD worldwide[4]. In view of the increasing number of patients with diabetes and hypertension, the incidence of CKD will likely rise even further.

The interactions between the intestinal microflora and the host maintain homeostasis under normal conditions[7]. There is a quantitative and qualitative alteration in the intestinal microflora in CKD patients[7]. Compared to the symbiotic flora, the pathogenic flora is increased[7]. The pathogenic gut microbiota produce uraemic toxins, particularly indoxyl sulphate (IS) and p-cresyl sulphate (PCS), which have been associated with increased inflammation and oxidative stress[7,8]. They greatly increase the risk of cardiovascular disease (CVD) and contribute to the progression and mortality of CKD[9,10].

Probiotics refer to living microorganisms which can colonise orimplant in the host gastrointestinal environment and exert beneficial health effects in the host[11,12].

For peer review only - http://bmjopen.bmj.com/site/about/guidelines.xhtml

Prebiotics are defined as a non-digestible food ingredient and can stimulate the growth and/or activity of one or a limited number of bacteria in the host intestines[11,12]. The combination of probiotics and prebiotics is defined as synbiotics[11,12].

In recent years, there has been a growing interest in reducing inflammation, improving kidney function and slowing the progression of CKD by restoring the symbiosis of gut microflora with probiotics, prebiotics or synbiotics in patients with CKD. A randomised trial suggested that synbiotics decrease serum PCS without reducing serum IS in non-dialysis CKD[13]. A pilot study suggested that probiotic dietary supplementation is more effective than placebo in reducing blood urea nitrogen (BUN) and improving quality of life in stage 3 or 4 CKD[14]. Another study showed that synbiotics had a beneficial effect on delaying CKD progression[9].

Guldris et al. performed a narrative review of the gut microbiota in chronic kidney disease in 2017[7]. There are some methodology shortcomings that may threaten the authenticity of their findings. First, there was insufficient literature retrieval without providing a comprehensive literature search strategy. Second, the 'risk of bias' of the included studies was not assessed using a validated tool. Third, they did not conduct statistical analysis and grade the evidence.

A systematic review suggests that prebiotic and probiotic therapy reduce IS and PCS in patients of end-stage kidney disease (ESKD) on haemodialysis[15]. However, whether the evidence is transferrable to all stages of CKD patients remains unclear. To our knowledge, the efficacy and safety of probiotics, prebiotics or sybiotics for non-dialysis or non-renal transplant patients with CKD has not been rigorously and systematically evaluated.

The objective of this study is to provide a comprehensive systematic review to collect and critically appraise all of the available evidence on the efficacy and safety of probiotics, prebiotics and synbiotics in the management of non-dialysis or non-renal transplant patients with CKD.

METHODS:

This protocol was developed following the Preferred Reporting Items for Systematic reviews and Meta-Analyses guidelines for protocols (PRISMA-P) [16].

Types of studies

We will include parallel-group and crossover randomised controlled trials (RCTs). Quasi-RCTs, controlled clinical trials (CCTs), controlled before and after trials, and cluster-RCTs will be excluded to avoid a biased estimate of treatment effects[12].

For peer review only - http://bmjopen.bmj.com/site/about/guidelines.xhtml

Types of participants

Patients with any stage of CKD (as defined by KDIGO) will be included[17]. We will consider patients of any age, both sexes, any ethnicity, and studies based in any clinical setting.

People with end-stage kidney disease (ESKD) treated with dialysis or kidney transplantation will be excluded. Normal people or non-CKD patients in control groups will be excluded.

Types of interventions

Experimental interventions

Probiotics, prebiotics and synbiotics at any dose, frequency, duration, route of delivery or setting, combination or preparation will be included[18].

Comparator interventions

Placebo, no treatment, and any other active pharmacological or non-pharmacological treatment (any dose, frequency, duration, route of delivery or setting, combination or preparation) will be included. We will exclude RCTs with probiotics, prebiotics and synbiotics in both arms.

Types of outcome measures:

Primary outcome

The primary outcome measure is uraemic toxins (including but not limited to PCS and serum IS).

Secondary outcomes

The secondary outcome measures are listed below:

- kidney function measures (including but not limited to blood urea nitrogen, glomerular filtration rate, creatinine clearance, serum creatinine);
- Major adverse cardiovascular events as defined by the investigators (including but not limited to myocardial infarction, coronary artery disease, heart failure, cerebrovascular disease and peripheral vascular disease);
- All-cause mortality and cause-specific death, such as cardiovascular mortality, sudden death, and infection-related mortality;
- Progression to ESKD requiring renal replacement therapy (RRT: haemodialysis, peritoneal dialysis or kidney transplantation);
- Quality of life measured by a validated scale, such as Kidney Disease Quality of Life (KDQoL);
- Study-related adverse events;

For peer review only - http://bmjopen.bmj.com/site/about/guidelines.xhtml

Search methods for identification of studies

Electronic searches

An extensive literature search will be undertaken to identify potentially eligible studies in the electronic databases PubMed, EMBASE, Web of Science, and Cochrane Central Register of Controlled Trials (CENTRAL).

The search strategy was developed by a medical librarian (JS) with ten years' experience as an information specialist according to search terms from previous literature reviews[17,18]. The search strategy for the PubMed database is attached (appendix 1). The search items are adjusted properly to suit other databases. No language restriction will be applied to the search.

Searching other resources

We will search ClinicalTrials.gov and the World Health Organization International Clinical Trials Registry Platform (ICTRP) to identify unpublished or ongoing studies. Google Scholar will be searched using key words to identify grey literature[19]. The reference lists of all retrieved studies and previous systematic reviews will be checked.

Data collection and analysis

Selection of studies

Two review authors (LW and BY) will independently run literature searches. All of the identified references from different electronic databases and other resources will be imported into EndNote software[20]. Duplicate records will be removed. Titles and abstracts will be screened to identify potentially eligible studies derived from the electronic search strategy. Then, full-text reports of all potentially relevant search results will be retrieved for the final selection of eligible studies. Disagreements will be resolved through consensus or consultation of a third reviewer (JZ).

Including duplicated data in a meta-analysis may lead to overestimation of intervention effects[21]. If uncertainties remain in justifying multiple publications from a single data set, review authors will contact the report authors for clarification[21].

A Preferred Reporting Items for Systematic Reviews and Meta-Analysis flow diagram will be drawn to present the screening process.

Data extraction and management

Two review authors (SH and HY) will independently extract the following data from all included studies using a standardised data extraction form. The form will be specifically designed and piloted by review authors. Any disagreement will be resolved through discussion. If a consensus can not be reached, a third reviewer (JZ) will be contacted to reach a decision.

For peer review only - http://bmjopen.bmj.com/site/about/guidelines.xhtml

- Study characteristics: title, authors, publication year, design, randomisation, concealed allocation, blinding, control, sample size, funding
- Participant characteristics: age, sex, number in each group, diagnostic criteria for CKD
- Intervention details: intervention treatment, comparator treatment, dose, route, number of patients in statistical analysis
- Outcome measures of interest in terms of efficacy and adverse events

Assessment of risk of bias in included studies

Two review authors (MP and JS) will independently assess the ‘risk of bias’ of each included study using the Cochrane ‘risk of bias’ tool in RevMan 5.3[21].

This tool includes seven core items: random sequence generation, allocation concealment, blinding of participants and personnel, blinding of outcome assessment, incomplete outcome data, selective reporting, and other potential sources of bias[21].

Each item is rated as a high, low or unclear risk of bias. The reviewers will grade the degree of ‘risk of bias’ for each item by their judgement.

Disagreements will be resolved through discussion. If necessary, a third reviewer (JZ) will be consulted to achieve consensus.

Study authors will be contacted to request further details if study information is insufficient.

We will present a ‘Risk of bias summary’ figure and ‘Risk of bias graph’ [21].

Measures of treatment effect

The risk ratio (RR) with its 95% confidence intervals (CIs) will be calculated for dichotomous outcomes.

The mean difference (MD) or standardised mean difference (SMD) with 95% CIs will be calculated for continuous outcomes.

The MD will be used when the same scale is used to measure an outcome. The SMD will be employed when different scales are used to measure the same outcome.

If a mixture of endpoint data and changes from baseline data for continuous outcomes are reported in included studies, subgroup analysis will be performed in which effect size estimates between subgroups are not combined[22].

A narrative description of results will be provided when the number of trials for the meta-analysis is less than two[22].

Unit of analysis issues

If crossover RCTs are included, we will only analyse the data from the first phase of the included study[23].

If studies include multiple treatment groups, review authors will assess which

For peer review only - http://bmjopen.bmj.com/site/about/guidelines.xhtml

intervention groups are relevant to the systematic review[21]. To overcome a unit-of-analysis error, combining intervention groups are recommended to create a single pair-wise comparison[21].

Dealing with missing data

Missing data may occur in any outcome of interest. If necessary, we will contact the authors of studies to clarify and obtain additional data. We will not fill missing data for any outcome in the primary analysis. However, we will assess the impact of missing data in the sensitivity analysis.

If the standard deviation (SD) is not reported, we will calculate the SD according to the data from the trial or study authors if possible.

Any potential impact of missing data will be discussed in the 'discussion' section of the full review.

Assessment of heterogeneity

We will assess the statistical heterogeneity by visual inspection of forest plots and a Chi-squared test. In addition, the I^2 statistical value will be calculated to quantify heterogeneity. We will categorise heterogeneity as follows:

- I^2 of 0%-40% as might not be important;
- I^2 of 30%-60% as may represent moderate heterogeneity;
- I^2 of 50%-90% as may represent substantial heterogeneity;
- I^2 of 75%-100% as considerable heterogeneity.

If the P-value from a Chi-squared test is less than 0.10 or $I^2 > 50\%$, the heterogeneity will be detected across studies[21,24]. We will assess potential clinical heterogeneity by prespecified subgroup analyses, which may partly explain the statistical heterogeneity.

Assessment of reporting bias

If at least 10 eligible studies are included in a meta-analysis, publication bias will be explored by assessing a funnel plot for signs of asymmetry[23]. If asymmetry is identified, possible reasons will be explored.

Data synthesis

An overall estimate of the intervention effect will be determined by combining more than one study with a meta-analysis when the same intervention and outcomes with comparable methods in a similar population are examined.

We plan to estimate the overall risk ratio (RR) using the Mantel-Haenszel method for dichotomous outcomes[23].

For continuous outcomes, the MD or SMD will be estimated based on the same

For peer review only - http://bmjopen.bmj.com/site/about/guidelines.xhtml

or different scales for measuring the same outcome.

If statistical heterogeneity is low, we will perform a meta-analysis with fixed-effect models. Otherwise, random-effect models will be used to provide a more conservative estimate of the difference.

We will provide a narrative summary of results from individual studies when it is impossible to conduct a meta-analysis.

Subgroup analysis and investigation of heterogeneity

We will perform subgroup analysis based on the type of intervention, if possible. All subgroup analysis results will be presented in forest plots.

When more than 80% of participants come from one or two trials of included studies, the fixed-effect method could provide a more conservative estimate of the difference[25]. These studies will be analysed as one of the subgroups[25].

If the heterogeneity is statistically significant ($P<0.10$), we will conduct a meta-regression analysis to investigate the heterogeneity.

Sensitivity analysis

We will perform sensitivity analyses to determine whether our findings are robust.

We plan to conduct sensitivity analysis for the following factors:

- Impact of model chosen: fixed-effect model versus random-effect model
- Risk of bias of included studies: excluding studies with high or unclear risk of bias
- Impact of trial design: excluding studies with a crossover design
- Impact of the missing data: excluding studies which lost over 50% of patients to follow-up

Summary of findings table

Two review authors (MP and JZ) will independently assess the quality of evidence for each outcome using the GRADE approach. Five factors (study limitations, imprecision, inconsistency, indirectness, and publication bias) could decrease the quality of evidence. The quality of evidence will fall into one of four categories from high to very low[26].

We will create 'summary of findings' tables for all outcomes using GRADEpro GDT[27]. They will include the number of studies, quality assessment, summary of findings, and importance of the outcome.

For peer review only - http://bmjopen.bmj.com/site/about/guidelines.xhtml

AMENDMENTS

We will provide the date, description, and rationale of any modification in the event of protocol amendments.

DISSEMINATION

This systematic review will provide a comprehensive assessment regarding the efficacy and safety of probiotics, prebiotics or synbiotics for non-dialysis or non-renal transplant patients with CKD. It will be fundamental for reliable recommendations in the management of CKD.

We will publish findings from this systematic review in a peer-reviewed scientific journal, and the data set will be made freely available.

Protocol registration: This protocol has been registered on PROSPERO (CRD42017079177).

Contributors:

JZ, MP and HY conceived the study. JZ, MP and HY provided general guidance to the drafting of the protocol. JZ and MP drafted the protocol. JS designed the search strategy. MP, LW, SH, BY, JS, HY and JZ drafted the manuscript. MP, LW, SH, BY, JS, HY and JZ reviewed and revised the manuscript. All authors have read and approved the final version of the manuscript.

Acknowledgements:

The authors thank Wei Mu for revising the English language of the manuscript.

Funding:

This study is supported by the National Natural Science Foundation of China [grant number 81703860] and the National Natural Science Foundation of China [grant number 81673909].

Competing interests: None declared.

Ethics approval: Not required.

Provenance and peer review: Not commissioned; externally peer reviewed.

Data sharing statement: We will publish findings from this systematic review in a peer-reviewed scientific journal, and data set will be made freely available.

For peer review only - http://bmjopen.bmj.com/site/about/guidelines.xhtml

Open access: This is an Open Access article distributed in accordance with the terms of the Creative Commons Attribution (CC BY 4.0) license, which permits others to distribute, remix, adapt and build upon this work, for commercial use, provided the original work is properly cited. See: http://creativecommons.org/licenses/by/4.0/

References:

[1] Luis Vitetta, Glenda Gobe. Uremia and chronic kidney disease: The role of the gutmicroflora and therapies with pro- and prebiotics. Mol Nutr Food Res 2013;57(5):824-32.

[2] Kidney Disease: Improving Global Outcomes (KDIGO) CKD WorkGroup. KDIGO clinical practice guideline for the evaluation and management of chronic kidney disease. Kidney Int Suppl. 2013;3:1-150.

[3] Paul E. Stevens, Adeera Levin, Kidney Disease: Improving Global Outcomes Chronic Kidney Disease Guideline Development Work Group Members. Evaluation and Management of Chronic Kidney Disease: Synopsis of the Kidney Disease: Improving Global Outcomes 2012 Clinical Practice Guideline. Ann Intern Med 2013;158(11):825-30.

[4] Angela C Webster, Evi V Nagler, Rachael L Morton, et al. Chronic kidney disease. Lancet 2017;389:1238-52.

[5] Vivekanand Jha, Guillermo Garcia-Garcia, Kunitoshi Iseki, et al. Chronic kidney disease: global dimension and perspectives. Lancet 2013;382(9888):260-72.

[6] Csaba P Kovesdy, Kamyar Kalantar-Zadeh. Enter the dragon: a Chinese epidemic of chronic kidney disease? Lancet 2012;379(9818):783-5.

[7] Secundino Cigarran Guldris, Emilio González Parra, Aleix Cases Amenós. Gut microbiota in chronic kidney disease. Mefrologia, 2017,37(1):9-19.

[8] Megan Rossi, David W. Johnson, Mark Morrison, et al. SYNbiotics Easing Renal failure by improving GutmicrobiologY (SYNERGY): a protocol of placebo-controlled randomised cross-over trial. BMC Nephrology 2014;15:106.

[9] B. Guida, R. Germanò, R. Trio, et al. Effect of short-term synbiotic treatment on plasma p-cresol levels in patients with chronic renal failure: A randomized clinical trial. Nutr Metab Cardiovasc Dis 2014;24(9):1043-9.

[10] Cheng-Jui Lin, Vincent Wu, Pei-Chen Wu, et al. Meta-Analysis of the Associations of p-Cresyl Sulfate (PCS) and Indoxyl Sulfate (IS) with Cardiovascular Events and All-Cause Mortality in Patients with Chronic Renal Failure. Plos one 2015;10(7):e0132589.

[11] Jürgen Schrezenmeir and Michael de Vrese. Probiotics, prebiotics, and synbiotics- approaching a definition. Am J Clin Nutr 2001;73(2 Suppl): 361S-364S.

[12] Lulong bo, jinbao li, tianzhu tao, et al. Probiotics for preventing ventilator-associated pneumonia. Cochrane Database Syst Rev 2014;10:CD009066.

For peer review only - http://bmjopen.bmj.com/site/about/guidelines.xhtml

[13] Megan Rossi, David W. Johnson, Mark Morrison, et al. Synbiotics Easing Renal Failure by Improving Gut Microbiology (SYNERGY): A Randomised Trial. Clin J Am Soc Nephrol 2016;11(2):223-31.

[14] Ranganathan N, Ranganathan P, Friedman EA, et al. Pilot Study of Probiotic Dietary Supplementation for Promoting Healthy Kidney Function in Patients with Chronic Kidney Disease. Adv Ther 2010;27(9):634-647.

[15] Megan Rossi, Kerenaftali Klein, David W. Johnson, et al. Pre-, Pro-, and Synbiotics: Do They Have a Role in Reducing Uremic Toxins? A Systematic Review and Meta-Analysis. Int J Nepphrol 2012;2012:673631.

[16] Larissa Shamseer, David Moher, Mike Clarke, et al. Preferred reporting items for systematic review and meta-analysis protocols (PRISMA-P) 2015: elaboration and explanation. BMJ 2015;350:g7647.

[17] Natale P, Ruospo M, Saglimbene VM, et al. Interventions for improving sleep quality in people with chronic kidney disease. Cochrane Database Syst Rev 2017;4:CD012625.

[18] Chowdhurg A, Varadhan K, Neal K, et al. Perioperative prebiotics, probiotics or synbiotics for elective abdominal surgery in adults. Cochrane Database Syst Rev 2011;7:CD009246.

[19] Theodosia Adom, Thandi Puoane, Anniza De Villiers, et al. Protocol for systematic review of school-based interventions to prevent and control obesity in African learners. BMJ Open 2017;7(3):e013540.

[20] Lorainne Tudor Car, Rifat Atun. eLearning for health system leadership and management capacity building: a protocol for a systematic review. BMJ Open 2017;7(8):e017050.

[21] Higgins JPT, Green S. Cochrane Handbook for Systematic Reviews of Interventions Version 5.1.0 (updated March 2011). The Cochrane Collaboration, 2011. http://www.handbook.cochrane.org.

[22] Bae S, Kamynina E, Farinola AF, et al. Provision of folic acid for reducing arsenic toxicity in arsenic-exposed children and adults. Cochrane Database Syst Rev 2017:5:CD012649.

[23] Aamann L, Dam G, Rinnov A, et al. Physical exercise for people with cirrhosis. Cochrane Database Syst Rev 2017:6:CD012678.

[24] Susanne Roehr, Alexander Pabst, Tobias Luck, et al. Secular trends in the incidence of dementia in high-income countries: a protocol of a systematic review and a planned meta-analysis. BMJ Open 2017;7(4):e013630.

[25] Browning GG, Howell P, Whitmer WM, et al. Unilateral versus bilateral hearing aids for bilateral hearing impairment in adults. Cochrane Database Syst Rev 2017:5:CD012665.

[26] Gordon Guyatt, Andrew D. Oxman, Elie Akl, et al. GRADE guidelines 1.

For peer review only - http://bmjopen.bmj.com/site/about/guidelines.xhtml

Introduction-GRADE evidence profiles and summary of findings tables. J Clin Epidemiol 2011;64(4):383-394.

[27] GRADEpro GDT. GRADEpro Guideline Development Tool. 2015. https://gradepro.org.

For peer review only

Appendix 1.

The search strategy for the Pubmed database:

1. Kidney Diseases/
2. Renal Insufficiency/
3. exp Renal Insufficiency, Chronic/
4. (kidney disease* or renal disease* or kidney failure or renal failure or kidney insufficienc* or renal insufficienc* or frasier syndrome).tw.
5. (ESRF or ESKF or ESRD or ESKD).tw.
6. (CKF or CKD or CRF or CRD).tw.
7. or/1-6
8. Probiotics/
9. probiotic*.tw.
10. Synbiotics/
11. synbiotic*.tw.
12. Prebiotics/
13. prebiotic*.tw.
14. exp Lactobacillus/
15. Lactobacillus.tw.
16. lactobacilli*.tw.
17. exp Bifidobacterium/
18. bifidobacteri*.tw.
19. exp Bacillus/
20. bacilli*.tw.
21. bacillu*.tw.
22. Clostridium butyricum/
23. clostridium butyricum.tw.
24. Streptococcus thermophilus/
25. streptococcus thermophill*.tw.
26. exp Escherichia coli/
27. (escherichia coli or E coli or EAggEC).tw.

For peer review only

28. exp Propionibacterium/
29. propionibacteria*.tw.
30. propionibacterium freudenreich*.tw.
31. exp Enterococcus faecium/
32. enterococcus faecium.tw.
33. streptococcus faecium.tw.
34. exp Saccharomyces/
35. (saccharomyces boulardi or Saccharomyces cerevisiae or Baker Yeast* or S cerevisiae or Bakers Yeast or Brewer* Yeast).tw.
36. vsl3.tw.
37. vsl 3.tw.
38. ganedenBc.tw.
39. lafti b94.tw.
40. mutaflor.tw.
41. actimel.tw.
42. cultura.tw.
43. yakult.tw.
44. l casei.tw.
45. shirota.tw.
46. Enterobacteriaceae/
47. proviva.tw.
48. vifit.tw.
49. verum.tw.
50. bio K.tw.
51. multibionta.tw.
52. oligofructose.mp
53. Inulin/
54. Inulin.tw
55. or/8-54
56. 7 and 55

For peer review only

57. randomized controlled trial.pt.
58. controlled clinical trial.pt.
59. randomised.tw.
60. randomized.tw.
61. placebo.tw.
62. randomly.tw.
63. trial.tw.
64. groups.tw.
65. or/57-64
66. exp animals/ not humans.sh.
67. 65 not 66
68. 56 and 67

For peer review only

PRISMA-P 2015 checklist

Section and topic	Item No	Page	Checklist item
Administrative information			
Title			
Identification	1a	1	Probiotics, prebiotics and synbiotics for chronic kidney disease: protocol for a systematic review and meta-analysis
Update	1b		No
Registration	2	2	This protocol has been registered on PROSPERO (CRD42017079177).
Authors:			
Contact	3a	1	Ming Pei[1], Lijuan Wei[2], Shouci Hu[2], Bo Yang[1], Jinhua Si[3], Hongtao Yang[1*], Jingbo Zhai[4*]<br>[1] Division of Nephrology, First Teaching Hospital of Tianjin University of Traditional Chinese Medicine, 314 Anshanxi Road, Nankai District, Tianjin 300193, China; [2] Graduate College, Tianjin University of Traditional Chinese Medicine, 312 Anshanxi Road, Nankai District, Tianjin 300193, China; [3]Library of Tianjin University of Traditional Chinese Medicine, 312 Anshanxi Road, Nankai District, Tianjin 300193, China; [4] Research institute of Traditional Chinese Medicine, Tianjin University of Traditional Chinese Medicine, 312 Anshanxi Road, Nankai District, Tianjin 300193, China.<br>Ming Pei and Lijuan Wei contributed equally.<br>Correspondence to Hongtao Yang, tjtcmht@126.com; Jingbo Zhai, zhaijingbo@foxmail.com
Contributions	3b	9	JZ, MP and HY conceived the study. JZ, MP and HY provided general guidance to the drafting of the protocol. JZ and MP drafted the protocol. JS designed the search strategy. MP, LW, SH, BY, JS, HY and JZ drafted the manuscript. MP, LW, SH, BY, JS, HY and JZ reviewed and revised the

For peer review only

			manuscript. All authors have read and approved the final version of the manuscript.
Amendments	4	9	We will provide the date, description, and rationale of any modification in the event of protocol amendments.
Support:			
Sources	5a	9	This study is supported by the National Natural Science Foundation of China [grant number 81703860] and the National Natural Science Foundation of China [grant number 81673909].
Sponsor	5b	9	JZ, MP and HY are sponsors.
Role of sponsor or funder	5c	9	MP and HY conceived the study. JZ, MP and HY provided general guidance to the drafting of the protocol. JZ and MP drafted the protocol. MP, HY and JZ drafted the manuscript. MP, HY and JZ reviewed and revised the manuscript.
Introduction			
Rationale	6	2-3	There is a growing interest in probiotics, prebiotics and synbiotics supplements for patients with chronic kidney disease (CKD). However, no comprehensive systematic review has been conducted to evaluate the efficacy and safety of probiotics, prebiotics or sybiotics for CKD patients without dialysis and renal transplantation.
Objectives	7	3	We aim to assess the efficacy and safety of probiotics, prebiotics or sybiotics for non-dialysis or non-renal transplant patients with CKD.
Methods			
Eligibility criteria	8	3-4	Types of studies<br>We will include parallel-group and crossover randomised controlled trials (RCTs). Quasi-RCTs, controlled clinical trials (CCTs), controlled before and after trials, and cluster-RCTs will be excluded to avoid a biased estimate of treatment effects.<br>Types of participants<br>Patients with any stage of CKD (as defined by KDIGO) will be included. We will consider

For peer review only

			patients of any age, both sexes, any ethnicity, and studies based in any clinical setting.<br>People with end-stage kidney disease (ESKD) treated with dialysis or kidney transplantation will be excluded. Normal people or non-CKD patients in control groups will be excluded.<br>Types of interventions<br>Experimental interventions<br>Probiotics, prebiotics and synbiotics at any dose, frequency, duration, route of delivery or setting, combination or preparation will be included.<br>Comparator interventions<br>Placebo, no treatment, and any other active pharmacological or non-pharmacological treatment (any dose, frequency, duration, route of delivery or setting, combination or preparation) will be included. We will exclude RCTs with probiotics, prebiotics and synbiotics in both arms.<br>Types of outcome measures:<br>Primary outcome<br>The primary outcome measure is uraemic toxins (including but not limited to PCS and serum IS).<br>Secondary outcomes<br>The secondary outcome measures are listed below:<br>● kidney function measures (including but not limited to blood urea nitrogen, glomerular filtration rate, creatinine clearance, serum creatinine);<br>● Major adverse cardiovascular events as defined by the investigators (including but not limited to myocardial infarction, coronary artery disease, heart failure, cerebrovascular disease and peripheral vascular disease);<br>● All-cause mortality and cause-specific death, such as cardiovascular mortality, sudden death, and infection-related mortality;<br>● Progression to ESKD requiring renal replacement therapy (RRT: haemodialysis, peritoneal

For peer review only

			dialysis or kidney transplantation);<br>● Quality of life measured by a validated scale, such as Kidney Disease Quality of Life (KDQoL);<br>● Study-related adverse events;
Information sources	9	5	An extensive literature search will be undertaken to identify potentially eligible studies in the electronic database of PubMed, EMBASE, Web of Science, and Cochrane Central Register of Controlled Trials (CENTRAL). We will search ClinicalTrials.gov and World Health Organization International Clinical Trials Registry Platform (ICTRP) to identify unpublished or ongoing studies. Google Scholar will be searched using key words to identify grey literature.
Search strategy	10	5	An extensive literature search will be undertaken to identify potentially eligible studies in the electronic database of Pubmed, EMBASE, Web of Science, and Cochrane Central Register of Controlled Trials (CENTRAL).<br>The search strategy is developed by a medical librarian (JS) with ten years' experience as an information specialist according to search terms from previous literature reviews. The search strategy for the PubMed database is attached (appendix 1). The search items are adjusted properly to suit other databases. No language restriction will be applied to the search.<br>We will search ClinicalTrials.gov and World Health Organization International Clinical Trials Registry Platform (ICTRP) to identify unpublished or ongoing studies. Google Scholar will be searched using key words to identify grey literature. The reference lists of all retrieved studies and previous systematic reviews will been checked.
Study records			
Data management	11a	5	All of the identified references from different electronic databases and other resources will be imported into the EndNote software. Duplicate records will be removed.
Selection process	11b	5	Two review authors (LW and BY) will independently run literature searches. All of identified references from different electronic databases and other resources will be imported into the EndNote

For peer review only

			software. Duplicate records will be removed. Titles and abstracts will be screened to identify potentially eligible studies derived from the electronic search strategy. Then, full-text reports of all potentially relevant search results will be retrieved for the final selection of eligible studies. Disagreements will be resolved through consensus or consultation of a third reviewer (JZ). Including duplicated data in a meta-analysis may lead to overestimation of intervention effects. If uncertainties remain in justifying multiple publications from a single data set, review authors will contact with report authors for the clarification. A Preferred Reporting Items for Systematic Reviews and Meta-Analysis flow diagram will be drawn to present the screening process.
Data collection process	11c	5	Two review authors (SH and HY) will independently extract the following data from all included studies using a standardised data extraction form. The form will be sepcifically designed and piloted by review authors. Any disagreement will be resolved through discussion. If a consensus could not be reached, a third reviewer (JZ) will be contacted to reach a decision.
Data items	12	6	Study characteristics: title, authors, publication year, design, randomization, concealed allocation, blinding, control, sample size, funding<br>Participant characteristics: age, sex, number in each group, diagnostic criteria for CKD<br>Intervention details: intervention treatment, comparator treatment, dose, route, number of patients in statistic analysis<br>Outcome measures of interest in terms of efficacy and adverse events
Outcomes and prioritization	13	4	Primary outcome:<br>The primary outcome measure is uraemic toxins (including but not limited to PCS and serum IS).<br>Secondary outcomes:<br>The secondary outcome measures are listed below:<br>● kidney function measures (including but not limited to blood urea nitrogen, glomerular filtration

For peer review only

			rate, creatinine clearance, serum creatinine);<br>● Major adverse cardiovascular events as defined by the investigators (including but not limited to myocardial infarction, coronary artery disease, heart failure, cerebrovascular disease and peripheral vascular disease);<br>● All-cause mortality and cause-specific death, such as cardiovascular mortality, sudden death, infection-related mortality;<br>● Progression to ESKD requiring renal replacement therapy (RRT: haemodialysis, peritoneal dialysis or kidney transplantation);<br>● Quality of life measured by a validated scale, such as Kidney Disease Quality of Life (KDQoL);<br>● Study-related adverse events;
Risk of bias in individual studies	14	6	Two review authors (MP and JS) will independently assess the 'risk of bias' of each included study using the Cochrane 'risk of bias' tool in RevMan 5.3.<br>This tool includes seven core items: random sequence generation, allocation concealment, blinding of participants and personnel, blinding of outcome assessment, incomplete outcome data, selective reporting, and other potential sources of bias.<br>Each item is rated as high, low or unclear risk of bias. The reviewers will grade the degree of 'risk of bias' for each item with their judgements.<br>Disagreements will be resolved through discussion. If necessary, a thrid reviewer (JZ) will be consulted to achieve consensus.<br>Study authors will be contacted to request further details if study infromation is insufficient.<br>We will present a 'Risk of bias summary' figure and 'Risk of bias graph' .
Data synthesis	15a	7	An overall estimate of the intervention effect will be determined by combining more than one study with meta-analysis when the same intervention and outcomes with comparable methods in a similar population are examined.

For peer review only

	15b	7-8	We plan to estimate the overall risk ratio (RR) using the Mantel-Haenszel method for dichotomous outcomes. For continuous outcomes, the MD or SMD will be estimated based on the same or different scales for measuring the same outcome.<br>If statistical heterogeneity is low, we will perform a meta-analysis with fixed-effect models. Otherwise, random-effect models will be used to provide a more conservative estimate of the difference.
	15c	8	We will perform subgroup analysis based on the type of intervention, if possible. All subgroup analysis results will be presented in forest plots.<br>When more than 80% of participants come from one or two trials of included studies, the fixed-effect method could provide a more conservative estimate of the difference. These studies will be analysed as one of the subgroups.<br>If the heterogeneity is statistically significant (P<0.10), we will conduct meta-regression analysis to investigate the heterogeneity.<br>We will perform sensitivity analyses to determine whether our findings are robust.<br>We plan to conduct sensitivity analysis for the following factors:<br>● Impact of model chosen: fixed-effect model versus random-effect model<br>● Risk of bias of included studies: excluding studies with high or unclear risk of bias<br>● Impact of trial design: excluding studies with crossover design<br>● Impact of the missing data: excluding studies which lost over 50% of patients to follow-up
	15d	8	We will provide a narrative summary of results from individual studies when it is impossible to conduct a meta-analysis.
Meta-bias(es)	16	7	If at least 10 eligible studies are included in a meta-analysis, publication bias will be explored by assessing funnel plot for signs of asymmetry. If asymmetry is identified, possible reasons will be

For peer review only

			explored.
Confidence in cumulative evidence	17	8	Two review authors (MP and JZ) will independently assess the quality of evidence for each outcome using the GRADE approach. Five factors (study limitations, imprecision, inconsistency, indirectness, and publication bias) could decrease the quality of evidence. The quality of evidence will fall into one of four categories from high to very low.<br>We will create 'summary of findings' tables for all outcomes using GRADEpro GDT. They will include the number of studies, quality assessment, summary of findings, and importance of the outcome.

For peer review only

BMJ Open

Probiotics, prebiotics and synbiotics for chronic kidney disease: protocol for a systematic review and meta-analysis

Journal:	*BMJ Open*
Manuscript ID	bmjopen-2017-020863.R1
Article Type:	Protocol
Date Submitted by the Author:	05-Apr-2018
Complete List of Authors:	Pei, Ming; First Teaching Hospital of Tianjin University of Traditional Chinese Medicine, Division of Nephrology<br>Wei, Lijuan; Tianjin University of Traditional Chinese Medicine, Graduate College<br>Hu, Shouci; Tianjin University of Traditional Chinese Medicine, Graduate College<br>Yang, Bo; First Teaching Hospital of Tianjin University of Traditional Chinese Medicine, Division of Nephrology<br>Si, Jinhua; Tianjin University of Traditional Chinese Medicine, Library<br>Yang, Hongtao; First Teaching Hospital of Tianjin University of Traditional Chinese Medicine, Division of Nephrology<br>Zhai, Jingbo; Tianjin University of Traditional Chinese Medicine, Research institute of Traditional Chinese Medicine
**Primary Subject Heading**:	Renal medicine
Secondary Subject Heading:	Renal medicine
Keywords:	Probiotics, prebiotics, synbiotics, chronic kidney disease, systematic review, meta-analysis

SCHOLARONE™
Manuscripts

Probiotics, prebiotics and synbiotics for chronic kidney disease: protocol for a systematic review and meta-analysis

Ming Pei[1], Lijuan Wei[2], Shouci Hu[2], Bo Yang[1], Jinhua Si[3], Hongtao Yang[1*], Jingbo Zhai[4*]

[1] Division of Nephrology, First Teaching Hospital of Tianjin University of Traditional Chinese Medicine,314 Anshanxi Road, Nankai District, Tianjin 300193, China;

[2] Graduate College, Tianjin University of Traditional Chinese Medicine, 312 Anshanxi Road, Nankai District, Tianjin 300193, China;

[3]Library, Tianjin University of Traditional Chinese Medicine, 312 Anshanxi Road, Nankai District, Tianjin 300193, China;

[4] Research institute of Traditional Chinese Medicine, Tianjin University of Traditional Chinese Medicine, 312 Anshanxi Road, Nankai District, Tianjin 300193, China;

Ming Pei and Lijuan Wei contributed equally.

Correspondence to Hongtao Yang, tjtcmht@126.com; Jingbo Zhai, zhaijingbo@foxmail.com

For peer review only

ABSTRACT

Introduction:

There is growing interest in probiotic, prebiotic and synbiotic supplements for patients with chronic kidney disease (CKD). However, no comprehensive systematic review has been conducted to evaluate the efficacy and safety of probiotics, prebiotics or sybiotics for CKD patients without dialysis and renal transplantation. We aim to assess the efficacy and safety of probiotics, prebiotics and sybiotics for non-dialysis or non-renal transplant patients with CKD.

Methods and analysis:

An extensive literature search will be undertaken to identify potentially eligible studies in the electronic databases PubMed (1946 to present), EMBASE (1974 to present), Web of Science (1900 to present), and Cochrane Central Register of Controlled Trials (CENTRAL, all years). No language restriction will be applied to the search. Parallel-group and crossover randomised controlled trials will be included. The 'risk of bias' of each included study will be assessed using the Cochrane 'risk of bias' tool. The primary outcome is uraemic toxins. The secondary outcomes include kidney function, adverse cardiovascular events, all-cause mortality and cause-specific death, progression to end-stage kidney disease, quality of life, gastrointestinal function, and adverse events. The data will be synthesised with appropriate statistical

For peer review only - http://bmjopen.bmj.com/site/about/guidelines.xhtml

methods. The quality of evidence for each outcome will be assessed using the GRADE approach.

Ethics and dissemination:

No primary data will be collected, so ethical approval is not required. We will publish findings from this systematic review in a peer-reviewed scientific journal, and the data set will be made freely available.

This protocol has been registered on PROSPERO (CRD42017079177).

Strengths and limitations of this study

- This is the first systematic review to investigate the efficacy and safety of probiotics, prebiotics or sybiotics for non-dialysis or non-renal transplant patients with chronic kidney disease (CKD).
- The search strategy was developed by a medical librarian with ten years' experience as an information specialist.
- This protocol was developed following the Preferred Reporting Items for Systematic reviews and Meta-Analyses guidelines for protocols (PRISMA-P).
- Study heterogeneity will likely pose challenges for the meta-analysis.

BACKGROUND

Chronic kidney disease (CKD) is one of the most common chronic diseases worldwide[1]. It was defined as abnormalities of kidney structure or function, present for more than 3 months, with implications for health by Kidney Disease: Improving Global Outcomes (KDIGO)[2]. KDIGO categorised the severity of CKD into five stages according to glomerular filtration rate (GFR) and three stages based on albuminuria[3].

The incidence of CKD is subject to large variation worldwide[4]. The prevalence of CKD is globally estimated to be 8-16%, with 10.8% in China and 13.1% in America[5,6]. Diabetes and hypertension are the main risk factors for CKD worldwide[4]. In view of the increasing number of patients with diabetes and hypertension, the incidence of CKD will likely rise even further.

The interactions between the intestinal microflora and the host maintain homeostasis under normal conditions[7]. There is a quantitative and qualitative alteration in the intestinal microflora in CKD patients[7]. Compared to the symbiotic flora, the pathogenic flora is increased[7]. The pathogenic gut microbiota produce uraemic toxins, particularly indoxyl sulphate (IS) and p-cresyl sulphate (PCS), which have been associated with increased inflammation and oxidative stress[7,8]. They greatly increase the risk of cardiovascular disease (CVD) and contribute to the progression and mortality of CKD[9,10].

For peer review only - http://bmjopen.bmj.com/site/about/guidelines.xhtml

For peer review only

Probiotics refer to living microorganisms which can colonise orimplant in the host gastrointestinal environment and exert beneficial health effects in the host[11,12]. Prebiotics are defined as a non-digestible food ingredient and can stimulate the growth and/or activity of one or a limited number of bacteria in the host intestines[11,12]. The combination of probiotics and prebiotics is defined as synbiotics[11,12].

In recent years, there has been a growing interest in reducing inflammation, improving kidney function and slowing the progression of CKD by restoring the symbiosis of gut microflora with probiotics, prebiotics or synbiotics in patients with CKD. A randomised trial suggested that synbiotics decrease serum PCS without reducing serum IS in non-dialysis CKD[13]. A pilot study suggested that probiotic dietary supplementation is more effective than placebo in reducing blood urea nitrogen (BUN) and improving quality of life in stage 3 or 4 CKD[14]. Another study showed that the synbiotic had a beneficial effect on delaying CKD progression[9].

Guldris et al. performed a narrative review of the gut microbiota in chronic kidney disease in 2017[7]. There are some methodology shortcomings that may threaten the authenticity of their findings. First, there was insufficient literature retrieval without providing a comprehensive literature search strategy. Second, the 'risk of bias' of the included studies was not assessed using a validated tool. Third, they did not conduct statistical analysis and grade the evidence.

A systematic review suggests that prebiotic and probiotic therapy reduce IS and PCS in patients of end-stage kidney disease (ESKD) on haemodialysis[15]. However, whether the evidence is transferrable to all stages of CKD patients remains unclear. To our knowledge, the efficacy and safety of probiotics, prebiotics or sybiotics for non-dialysis or non-renal transplant patients with CKD has not been rigorously and systematically evaluated.

The objective of this study is to provide a comprehensive systematic review to collect and critically appraise all of the available evidence on the efficacy and safety of probiotics, prebiotics and synbiotics in the management of non-dialysis or non-renal transplant patients with CKD.

METHODS:

This protocol was developed following the Preferred Reporting Items for Systematic reviews and Meta-Analyses guidelines for protocols (PRISMA-P) [16].

Types of studies

We will include parallel-group and crossover randomised controlled trials (RCTs). Quasi-RCTs, controlled clinical trials (CCTs), controlled before and after trials, and cluster-RCTs will be excluded to avoid a biased estimate of treatment effects[12].

For peer review only - http://bmjopen.bmj.com/site/about/guidelines.xhtml

Types of participants

Non-dialysis or non-renal transplant patients in any stage of CKD (as defined by KDIGO) will be included[17]. We will consider patients of any age, both sexes, any ethnicity, and studies in any clinical setting. Normal people or non-CKD patients in control groups will be excluded.

Types of interventions

Experimental interventions

Probiotics, prebiotics and synbiotics at any dose, frequency, duration, route of delivery or setting, combination or preparation will be included[18].

Comparator interventions

Placebo, no treatment, and any other active pharmacological or non-pharmacological treatment (any dose, frequency, duration, route of delivery or setting, combination or preparation) will be included. We will exclude RCTs with probiotics, prebiotics and synbiotics in both arms.

Types of outcome measures:

Primary outcome

The primary outcome measure is uraemic toxins (including but not limited to phenols and indoles). Phenols include p-cresol, p-cresyl sulphate (PCS), p-cresyl glucuronide, et al[7]. Indoles include indoxyl sulfate (IS), indoleacetic acid, et al[7].

Secondary outcomes

The secondary outcome measures are listed below:

- kidney function measures (including but not limited to blood urea nitrogen, glomerular filtration rate, creatinine clearance, serum creatinine);
- Major adverse cardiovascular events as defined by the investigators (including but not limited to myocardial infarction, coronary artery disease, heart failure, cerebrovascular disease and peripheral vascular disease);
- All-cause mortality and cause-specific death, such as cardiovascular mortality, sudden death, and infection-related mortality;
- Progression to ESKD requiring renal replacement therapy (RRT: haemodialysis, peritoneal dialysis or kidney transplantation);
- Quality of life measured by a validated scale, such as Kidney Disease Quality of Life (KDQoL);
- Gastrointestinal (GI) function (including but not limited to the improvement of GI symptoms, transit time, GI tolerance);
- Study-related adverse events;

For peer review only - http://bmjopen.bmj.com/site/about/guidelines.xhtml

Search methods for identification of studies

Electronic searches

An extensive literature search will be undertaken to identify potentially eligible studies in the electronic databases PubMed (1946 to present), EMBASE (1974 to present), Web of Science (1900 to present), and Cochrane Central Register of Controlled Trials (CENTRAL, all years).

The search strategy was developed by a medical librarian (JS) with ten years' experience as an information specialist according to search terms from previous literature reviews[17,18]. The search strategy for the PubMed database is attached (appendix 1). The search items are adjusted properly to suit other databases. No language restriction will be applied to the search.

Searching other resources

We will search ClinicalTrials.gov and the World Health Organization International Clinical Trials Registry Platform (ICTRP) to identify unpublished or ongoing studies. Google Scholar will be searched using key words to identify grey literature[19]. The reference lists of all retrieved studies and previous systematic reviews will be checked.

Data collection and analysis

Selection of studies

Two review authors (LW and BY) will independently run literature searches. All of the identified references from different electronic databases and other resources will be imported into EndNote software[20]. Duplicate records will be removed. Titles and abstracts will be screened to identify potentially eligible studies derived from the electronic search strategy. Then, full-text reports of all potentially relevant search results will be retrieved for the final selection of eligible studies. Disagreements will be resolved through consensus or consultation of a third reviewer (JZ).

Including duplicated data in a meta-analysis may lead to overestimation of intervention effects[21]. If uncertainties remain in justifying multiple publications from a single data set, review authors will contact the report authors for clarification[21].

A Preferred Reporting Items for Systematic Reviews and Meta-Analysis flow diagram will be drawn to present the screening process.

Data extraction and management

Two review authors (SH and HY) will independently extract the following data from all included studies using a standardised data extraction form. The form will be specifically designed and piloted by review authors. Any disagreement will be resolved through discussion. If a consensus can not be reached, a third reviewer (JZ)

For peer review only - http://bmjopen.bmj.com/site/about/guidelines.xhtml

will be contacted to reach a decision.

- Study characteristics: title, authors, publication year, design, randomisation, concealed allocation, blinding, control, sample size, funding
- Participant characteristics: age, sex, number in each group, diagnostic criteria for CKD
- Intervention details: intervention treatment, comparator treatment, dose, route, number of patients in statistical analysis
- Outcome measures of interest in terms of efficacy and adverse events

Assessment of risk of bias in included studies

Two review authors (MP and JS) will independently assess the 'risk of bias' of each included study using the Cochrane 'risk of bias' tool in RevMan 5.3[21].

This tool includes seven core items: random sequence generation, allocation concealment, blinding of participants and personnel, blinding of outcome assessment, incomplete outcome data, selective reporting, and other potential sources of bias[21].

Each item is rated as a high, low or unclear risk of bias. The reviewers will grade the degree of 'risk of bias' for each item by their judgement.

Disagreements will be resolved through discussion. If necessary, a third reviewer (JZ) will be consulted to achieve consensus.

Study authors will be contacted to request further details if study information is insufficient.

We will present a 'Risk of bias summary' figure and 'Risk of bias graph' [21].

Measures of treatment effect

The risk ratio (RR) with its 95% confidence intervals (CIs) will be calculated for dichotomous outcomes.

The mean difference (MD) or standardised mean difference (SMD) with 95% CIs will be calculated for continuous outcomes.

The MD will be used when the same scale is used to measure an outcome. The SMD will be employed when different scales are used to measure the same outcome.

If a mixture of endpoint data and changes from baseline data for continuous outcomes are reported in included studies, subgroup analysis will be performed in which effect size estimates between subgroups are not combined[22].

A narrative description of results will be provided when the number of trials for the meta-analysis is less than two[22].

Unit of analysis issues

If crossover RCTs are included, we will only analyse the data from the first phase of the included study[23].

For peer review only - http://bmjopen.bmj.com/site/about/guidelines.xhtml

If studies include multiple treatment groups, review authors will assess which intervention groups are relevant to the systematic review[21]. To overcome a unit-of-analysis error, combining intervention groups are recommended to create a single pair-wise comparison[21].

Dealing with missing data

Missing data may occur in any outcome of interest. If necessary, we will contact the authors of studies to clarify and obtain additional data. We will not fill missing data for any outcome in the primary analysis. However, we will assess the impact of missing data in the sensitivity analysis.

If the standard deviation (SD) is not reported, we will calculate the SD according to the data from the trial or study authors if possible.

Any potential impact of missing data will be discussed in the 'discussion' section of the full review.

Assessment of heterogeneity

We will assess the statistical heterogeneity by visual inspection of forest plots and a Chi-squared test. In addition, the I^2 statistical value will be calculated to quantify heterogeneity. We will categorise heterogeneity as follows:

- I^2 of 0%-40% as might not be important;
- I^2 of 30%-60% as may represent moderate heterogeneity;
- I^2 of 50%-90% as may represent substantial heterogeneity;
- I^2 of 75%-100% as considerable heterogeneity.

If the P-value from a Chi-squared test is less than 0.10 or $I^2 > 50\%$, the heterogeneity will be detected across studies[21,24]. We will assess potential clinical heterogeneity by prespecified subgroup analyses, which may partly explain the statistical heterogeneity.

Assessment of reporting bias

If at least 10 eligible studies are included in a meta-analysis, publication bias will be explored by assessing a funnel plot for signs of asymmetry[23]. If asymmetry is identified, possible reasons will be explored.

Data synthesis

An overall estimate of the intervention effect will be determined by combining more than one study with a meta-analysis when the same intervention and outcomes with comparable methods in a similar population are examined.

We plan to estimate the overall risk ratio (RR) using the Mantel-Haenszel method for dichotomous outcomes[23].

For peer review only - http://bmjopen.bmj.com/site/about/guidelines.xhtml

For continuous outcomes, the MD or SMD will be estimated based on the same or different scales for measuring the same outcome.

If statistical heterogeneity is low, we will perform a meta-analysis with fixed-effect models. Otherwise, random-effect models will be used to provide a more conservative estimate of the difference.

We will provide a narrative summary of results from individual studies when it is impossible to conduct a meta-analysis.

Subgroup analysis and investigation of heterogeneity

We will perform subgroup analysis based on the type of intervention, if possible. All subgroup analysis results will be presented in forest plots.

When more than 80% of participants come from one or two trials of included studies, the fixed-effect method could provide a more conservative estimate of the difference[25]. These studies will be analysed as one of the subgroups[25].

If the heterogeneity is statistically significant ($P<0.10$), we will conduct a meta-regression analysis to investigate the heterogeneity.

Sensitivity analysis

We will perform sensitivity analyses to determine whether our findings are robust.

We plan to conduct sensitivity analysis for the following factors:

- Impact of model chosen: fixed-effect model versus random-effect model
- Risk of bias of included studies: excluding studies with high or unclear risk of bias
- Impact of trial design: excluding studies with a crossover design
- Impact of the missing data: excluding studies which lost over 50% of patients to follow-up

Summary of findings table

Two review authors (MP and JZ) will independently assess the quality of evidence for each outcome using the GRADE approach. Five factors (study limitations, imprecision, inconsistency, indirectness, and publication bias) could decrease the quality of evidence. The quality of evidence will fall into one of four categories from high to very low[26].

We will create ‘summary of findings’ tables for all outcomes using GRADEpro GDT[27]. They will include the number of studies, quality assessment, summary of findings, and importance of the outcome.

For peer review only - http://bmjopen.bmj.com/site/about/guidelines.xhtml

Patient and Public Involvement

No patients were involved in setting the research question or outcome measures. No patients were involved in developing plans for desgin or implementation of the study. There are no plans to disseminate the results of the research to study participants or the relevant patient community.

AMENDMENTS

We will provide the date, description, and rationale of any modification in the event of protocol amendments.

DISSEMINATION

This systematic review will provide a comprehensive assessment regarding the efficacy and safety of probiotics, prebiotics or synbiotics for non-dialysis or non-renal transplant patients with CKD. It will be fundamental for reliable recommendations in the management of CKD.

We will publish findings from this systematic review in a peer-reviewed scientific journal, and the data set will be made freely available.

Protocol registration: This protocol has been registered on PROSPERO (CRD42017079177).

Contributors:

JZ, MP and HY conceived the study. JZ, MP and HY provided general guidance to the drafting of the protocol. JZ and MP drafted the protocol. JS designed the search strategy. MP, LW, SH, BY, JS, HY and JZ drafted the manuscript. MP, LW, SH, BY, JS, HY and JZ reviewed and revised the manuscript. All authors have read and approved the final version of the manuscript.

Acknowledgements:

The authors thank Wei Mu for revising the English language of the manuscript.

Funding:

This study is supported by the National Natural Science Foundation of China [grant number 81703860] and the National Natural Science Foundation of China [grant number 81673909].

Competing interests: None declared.

Ethics approval: Not required.

For peer review only - http://bmjopen.bmj.com/site/about/guidelines.xhtml

Provenance and peer review: Not commissioned; externally peer reviewed.

Data sharing statement: We will publish findings from this systematic review in a peer-reviewed scientific journal, and data set will be made freely available.

Open access: This is an Open Access article distributed in accordance with the terms of the Creative Commons Attribution (CC BY 4.0) license, which permits others to distribute, remix, adapt and build upon this work, for commercial use, provided the original work is properly cited. See: http://creativecommons.org/licenses/by/4.0/

References:

[1] Luis Vitetta, Glenda Gobe. Uremia and chronic kidney disease: The role of the gutmicroflora and therapies with pro- and prebiotics. Mol Nutr Food Res 2013;57(5):824-32.

[2] Kidney Disease: Improving Global Outcomes (KDIGO) CKD WorkGroup. KDIGO clinical practice guideline for the evaluation and management of chronic kidney disease. Kidney Int Suppl. 2013;3:1-150.

[3] Paul E. Stevens, Adeera Levin, Kidney Disease: Improving Global Outcomes Chronic Kidney Disease Guideline Development Work Group Members. Evaluation and Management of Chronic Kidney Disease: Synopsis of the Kidney Disease: Improving Global Outcomes 2012 Clinical Practice Guideline. Ann Intern Med 2013;158(11):825-30.

[4] Angela C Webster, Evi V Nagler, Rachael L Morton, et al. Chronic kidney disease. Lancet 2017;389:1238-52.

[5] Vivekanand Jha, Guillermo Garcia-Garcia, Kunitoshi Iseki, et al. Chronic kidney disease: global dimension and perspectives. Lancet 2013;382(9888):260-72.

[6] Csaba P Kovesdy, Kamyar Kalantar-Zadeh. Enter the dragon: a Chinese epidemic of chronic kidney disease? Lancet 2012;379(9818):783-5.

[7] Secundino Cigarran Guldris, Emilio González Parra, Aleix Cases Amenós. Gut microbiota in chronic kidney disease. Mefrologia, 2017,37(1):9-19.

[8] Megan Rossi, David W. Johnson, Mark Morrison, et al. SYNbiotics Easing Renal failure by improving GutmicrobiologY (SYNERGY): a protocol of placebo-controlled randomised cross-over trial. BMC Nephrology 2014;15:106.

[9] B. Guida, R. Germanò, R. Trio, et al. Effect of short-term synbiotic treatment on plasma p-cresol levels in patients with chronic renal failure: A randomized clinical trial. Nutr Metab Cardiovasc Dis 2014;24(9):1043-9.

[10] Cheng-Jui Lin, Vincent Wu, Pei-Chen Wu, et al. Meta-Analysis of the Associations of p-Cresyl Sulfate (PCS) and Indoxyl Sulfate (IS) with Cardiovascular

For peer review only - http://bmjopen.bmj.com/site/about/guidelines.xhtml

Events and All-Cause Mortality in Patients with Chronic Renal Failure. Plos one 2015;10(7):e0132589.

[11] Jürgen Schrezenmeir and Michael de Vrese. Probiotics, prebiotics, and synbiotics- approaching a definition. Am J Clin Nutr 2001;73(2 Suppl): 361S-364S.

[12] Lulong bo, jinbao li, tianzhu tao, et al. Probiotics for preventing ventilator-associated pneumonia. Cochrane Database Syst Rev 2014;10:CD009066.

[13] Megan Rossi, David W. Johnson, Mark Morrison, et al. Synbiotics Easing Renal Failure by Improving Gut Microbiology (SYNERGY): A Randomised Trial. Clin J Am Soc Nephrol 2016;11(2):223-31.

[14] Ranganathan N, Ranganathan P, Friedman EA, et al. Pilot Study of Probiotic Dietary Supplementation for Promoting Healthy Kidney Function in Patients with Chronic Kidney Disease. Adv Ther 2010;27(9):634-647.

[15] Megan Rossi, Kerenaftali Klein, David W. Johnson, et al. Pre-, Pro-, and Synbiotics: Do They Have a Role in Reducing Uremic Toxins? A Systematic Review and Meta-Analysis. Int J Nepphrol 2012;2012:673631.

[16] Larissa Shamseer, David Moher, Mike Clarke, et al. Preferred reporting items for systematic review and meta-analysis protocols (PRISMA-P) 2015: elaboration and explanation. BMJ 2015;350:g7647.

[17] Natale P, Ruospo M, Saglimbene VM, et al. Interventions for improving sleep quality in people with chronic kidney disease. Cochrane Database Syst Rev 2017;4:CD012625.

[18] Chowdhurg A, Varadhan K, Neal K, et al. Perioperative prebiotics, probiotics or synbiotics for elective abdominal surgery in adults. Cochrane Database Syst Rev 2011;7:CD009246.

[19] Theodosia Adom, Thandi Puoane, Anniza De Villiers, et al. Protocol for systematic review of school-based interventions to prevent and control obesity in African learners. BMJ Open 2017;7(3):e013540.

[20] Lorainne Tudor Car, Rifat Atun. eLearning for health system leadership and management capacity building: a protocol for a systematic review. BMJ Open 2017;7(8):e017050.

[21] Higgins JPT, Green S. Cochrane Handbook for Systematic Reviews of Interventions Version 5.1.0 (updated March 2011). The Cochrane Collaboration, 2011. http://www.handbook.cochrane.org.

[22] Bae S, Kamynina E, Farinola AF, et al. Provision of folic acid for reducing arsenic toxicity in arsenic-exposed children and adults. Cochrane Database Syst Rev 2017:5:CD012649.

[23] Aamann L, Dam G, Rinnov A, et al. Physical exercise for people with cirrhosis. Cochrane Database Syst Rev 2017:6:CD012678.

[24] Susanne Roehr, Alexander Pabst, Tobias Luck, et al. Secular trends in the

For peer review only - http://bmjopen.bmj.com/site/about/guidelines.xhtml

incidence of dementia in high-income countries: a protocol of a systematic review and a planned meta-analysis. BMJ Open 2017;7(4):e013630.
[25] Browning GG, Howell P, Whitmer WM, et al. Unilateral versus bilateral hearing aids for bilateral hearing impairment in adults. Cochrane Database Syst Rev 2017:5:CD012665.
[26] Gordon Guyatt, Andrew D. Oxman, Elie Akl, et al. GRADE guidelines 1. Introduction-GRADE evidence profiles and summary of findings tables. J Clin Epidemiol 2011;64(4):383-394.
[27] GRADEpro GDT. GRADEpro Guideline Development Tool. 2015. https://gradepro.org.

For peer review only

Appendix 1.

The search strategy for the Pubmed database:

1. Kidney Diseases/
2. Renal Insufficiency/
3. exp Renal Insufficiency, Chronic/
4. (kidney disease* or renal disease* or kidney failure or renal failure or kidney insufficienc* or renal insufficienc* or frasier syndrome).tw.
5. (ESRF or ESKF or ESRD or ESKD).tw.
6. (CKF or CKD or CRF or CRD).tw.
7. or/1-6
8. Probiotics/
9. probiotic*.tw.
10. Synbiotics/
11. synbiotic*.tw.
12. Prebiotics/
13. prebiotic*.tw.
14. exp Lactobacillus/
15. Lactobacillus.tw.
16. lactobacilli*.tw.
17. exp Bifidobacterium/
18. bifidobacteri*.tw.
19. exp Bacillus/
20. bacilli*.tw.
21. bacillu*.tw.
22. Clostridium butyricum/
23. clostridium butyricum.tw.
24. Streptococcus thermophilus/
25. streptococcus thermophill*.tw.
26. exp Escherichia coli/
27. (escherichia coli or E coli or EAggEC).tw.

For peer review only

28. exp Propionibacterium/
29. propionibacteria*.tw.
30. propionibacterium freudenreich*.tw.
31. exp Enterococcus faecium/
32. enterococcus faecium.tw.
33. streptococcus faecium.tw.
34. exp Saccharomyces/
35. (saccharomyces boulardi or Saccharomyces cerevisiae or Baker Yeast* or S cerevisiae or Bakers Yeast or Brewer* Yeast).tw.
36. vsl3.tw.
37. vsl 3.tw.
38. ganedenBc.tw.
39. lafti b94.tw.
40. mutaflor.tw.
41. actimel.tw.
42. cultura.tw.
43. yakult.tw.
44. l casei.tw.
45. shirota.tw.
46. Enterobacteriaceae/
47. proviva.tw.
48. vifit.tw.
49. verum.tw.
50. bio K.tw.
51. multibionta.tw.
52. oligofructose.mp
53. Inulin/
54. Inulin.tw
55. or/8-54
56. 7 and 55

For peer review only

57. randomized controlled trial.pt.
58. controlled clinical trial.pt.
59. randomised.tw.
60. randomized.tw.
61. placebo.tw.
62. randomly.tw.
63. trial.tw.
64. groups.tw.
65. or/57-64
66. exp animals/ not humans.sh.
67. 65 not 66
68. 56 and 67

For peer review only

PRISMA-P 2015 checklist

Section and topic	Item No	Page	Checklist item
Administrative information			
Title			
Identification	1a	1	Probiotics, prebiotics and synbiotics for chronic kidney disease: protocol for a systematic review and meta-analysis
Update	1b		No
Registration	2	2	This protocol has been registered on PROSPERO (CRD42017079177).
Authors:			
Contact	3a	1	Ming Pei[1], Lijuan Wei[2], Shouci Hu[2], Bo Yang[1], Jinhua Si[3], Hongtao Yang[1*], Jingbo Zhai[4*] [1] Division of Nephrology, First Teaching Hospital of Tianjin University of Traditional Chinese Medicine, 314 Anshanxi Road, Nankai District, Tianjin 300193, China; [2] Graduate College, Tianjin University of Traditional Chinese Medicine, 312 Anshanxi Road, Nankai District, Tianjin 300193, China; [3]Library of Tianjin University of Traditional Chinese Medicine, 312 Anshanxi Road, Nankai District, Tianjin 300193, China; [4] Research institute of Traditional Chinese Medicine, Tianjin University of Traditional Chinese Medicine, 312 Anshanxi Road, Nankai District, Tianjin 300193, China.<br>Ming Pei and Lijuan Wei contributed equally.<br>Correspondence to Hongtao Yang, tjtcmht@126.com; Jingbo Zhai, zhaijingbo@foxmail.com
Contributions	3b	9	JZ, MP and HY conceived the study. JZ, MP and HY provided general guidance to the drafting of the protocol. JZ and MP drafted the protocol. JS designed the search strategy. MP, LW, SH, BY, JS, HY and JZ drafted the manuscript. MP, LW, SH, BY, JS, HY and JZ reviewed and revised the

For peer review only

			manuscript. All authors have read and approved the final version of the manuscript.
Amendments	4	9	We will provide the date, description, and rationale of any modification in the event of protocol amendments.
Support:			
Sources	5a	9	This study is supported by the National Natural Science Foundation of China [grant number 81703860] and the National Natural Science Foundation of China [grant number 81673909].
Sponsor	5b	9	JZ, MP and HY are sponsors.
Role of sponsor or funder	5c	9	MP and HY conceived the study. JZ, MP and HY provided general guidance to the drafting of the protocol. JZ and MP drafted the protocol. MP, HY and JZ drafted the manuscript. MP, HY and JZ reviewed and revised the manuscript.
Introduction			
Rationale	6	2-3	There is a growing interest in probiotics, prebiotics and synbiotics supplements for patients with chronic kidney disease (CKD). However, no comprehensive systematic review has been conducted to evaluate the efficacy and safety of probiotics, prebiotics or sybiotics for CKD patients without dialysis and renal transplantation.
Objectives	7	3	We aim to assess the efficacy and safety of probiotics, prebiotics or sybiotics for non-dialysis or non-renal transplant patients with CKD.
Methods			
Eligibility criteria	8	3-4	Types of studies<br>We will include parallel-group and crossover randomised controlled trials (RCTs). Quasi-RCTs, controlled clinical trials (CCTs), controlled before and after trials, and cluster-RCTs will be excluded to avoid a biased estimate of treatment effects.<br>Types of participants<br>Non-dialysis or non-renal transplant patients in any stage of CKD (as defined by KDIGO) will be

For peer review only

			included. We will consider patients of any age, both sexes, any ethnicity, and studies based in any clinical setting.<br>People with end-stage kidney disease (ESKD) treated with dialysis or kidney transplantation will be excluded. Normal people or non-CKD patients in control groups will be excluded.<br>Types of interventions<br>Experimental interventions<br>Probiotics, prebiotics and synbiotics at any dose, frequency, duration, route of delivery or setting, combination or preparation will be included.<br>Comparator interventions<br>Placebo, no treatment, and any other active pharmacological or non-pharmacological treatment (any dose, frequency, duration, route of delivery or setting, combination or preparation) will be included. We will exclude RCTs with probiotics, prebiotics and synbiotics in both arms.<br>Types of outcome measures:<br>Primary outcome<br>The primary outcome measure is uraemic toxins (including but not limited to phenols and indoles). Phenols include p-cresol, p-cresyl sulphate (PCS), p-cresyl glucuronide, et al[7]. Indoles include indoxyl sulfate (IS), indoleacetic acid, et al.<br>Secondary outcomes<br>The secondary outcome measures are listed below:<br>● kidney function measures (including but not limited to blood urea nitrogen, glomerular filtration rate, creatinine clearance, serum creatinine);<br>● Major adverse cardiovascular events as defined by the investigators (including but not limited to myocardial infarction, coronary artery disease, heart failure, cerebrovascular disease and

For peer review only

			peripheral vascular disease);<br>● All-cause mortality and cause-specific death, such as cardiovascular mortality, sudden death, and infection-related mortality;<br>● Progression to ESKD requiring renal replacement therapy (RRT: haemodialysis, peritoneal dialysis or kidney transplantation);<br>● Quality of life measured by a validated scale, such as Kidney Disease Quality of Life (KDQoL);<br>● Gastrointestinal (GI) function (including but not limited to the improvement of GI symptoms, transit time, GI tolerance);<br>● Study-related adverse events;
Information sources	9	5	An extensive literature search will be undertaken to identify potentially eligible studies in the electronic databases PubMed (1946 to present), EMBASE (1974 to present), Web of Science (1900 to present), and Cochrane Central Register of Controlled Trials (CENTRAL, all years). We will search ClinicalTrials.gov and World Health Organization International Clinical Trials Registry Platform (ICTRP) to identify unpublished or ongoing studies. Google Scholar will be searched using key words to identify grey literature.
Search strategy	10	5	An extensive literature search will be undertaken to identify potentially eligible studies in the electronic databases PubMed (1946 to present), EMBASE (1974 to present), Web of Science (1900 to present), and Cochrane Central Register of Controlled Trials (CENTRAL, all years).<br>The search strategy is developed by a medical librarian (JS) with ten years' experience as an information specialist according to search terms from previous literature reviews. The search strategy for the PubMed database is attached (appendix 1). The search items are adjusted properly to suit other databases. No language restriction will be applied to the search.<br>We will search ClinicalTrials.gov and World Health Organization International Clinical Trials Registry Platform (ICTRP) to identify unpublished or ongoing studies. Google Scholar will be

For peer review only

			searched using key words to identify grey literature. The reference lists of all retrieved studies and previous systematic reviews will been checked.
Study records			
Data management	11a	5	All of the identified references from different electronic databases and other resources will be imported into the EndNote software. Duplicate records will be removed.
Selection process	11b	5	Two review authors (LW and BY) will independently run literature searches. All of identified references from different electronic databases and other resources will be imported into the EndNote software. Duplicate records will be removed. Titles and abstracts will be screened to identify potentially eligible studies derived from the electronic search strategy. Then, full-text reports of all potentially relevant search results will be retrieved for the final selection of eligible studies. Disagreements will be resolved through consensus or consultation of a third reviewer (JZ). Including duplicated data in a meta-analysis may lead to overestimation of intervention effects. If uncertainties remain in justifying multiple publications from a single data set, review authors will contact with report authors for the clarification. A Preferred Reporting Items for Systematic Reviews and Meta-Analysis flow diagram will be drawn to present the screening process.
Data collection process	11c	5	Two review authors (SH and HY) will independently extract the following data from all included studies using a standardised data extraction form. The form will be sepcifically designed and piloted by review authors. Any disagreement will be resolved through discussion. If a consensus could not be reached, a third reviewer (JZ) will be contacted to reach a decision.
Data items	12	6	Study characteristics: title, authors, publication year, design, randomization, concealed allocation, blinding, control, sample size, funding<br>Participant characteristics: age, sex, number in each group, diagnostic criteria for CKD<br>Intervention details: intervention treatment, comparator treatment, dose, route, number of patients in

For peer review only

			statistic analysis<br>Outcome measures of interest in terms of efficacy and adverse events
Outcomes and prioritization	13	4	Primary outcome:<br>The primary outcome measure is uraemic toxins (including but not limited to phenols and indoles). Phenols include p-cresol, p-cresyl sulphate (PCS), p-cresyl glucuronide, et al[7]. Indoles include indoxyl sulfate (IS), indoleacetic acid, et al.<br>Secondary outcomes:<br>The secondary outcome measures are listed below:<br>● kidney function measures (including but not limited to blood urea nitrogen, glomerular filtration rate, creatinine clearance, serum creatinine);<br>● Major adverse cardiovascular events as defined by the investigators (including but not limited to myocardial infarction, coronary artery disease, heart failure, cerebrovascular disease and peripheral vascular disease);<br>● All-cause mortality and cause-specific death, such as cardiovascular mortality, sudden death, infection-related mortality;<br>● Progression to ESKD requiring renal replacement therapy (RRT: haemodialysis, peritoneal dialysis or kidney transplantation);<br>● Quality of life measured by a validated scale, such as Kidney Disease Quality of Life (KDQoL);<br>● Study-related adverse events;
Risk of bias in individual studies	14	6	Two review authors (MP and JS) will independently assess the 'risk of bias' of each included study using the Cochrane 'risk of bias' tool in RevMan 5.3.<br>This tool includes seven core items: random sequence generation, allocation concealment, blinding of participants and personnel, blinding of outcome assessment, incomplete outcome data, selective reporting, and other potential sources of bias.

For peer review only

			Each item is rated as high, low or unclear risk of bias. The reviewers will grade the degree of 'risk of bias' for each item with their judgements.<br>Disagreements will be resolved through discussion. If necessary, a thrid reviewer (JZ) will be consulted to achieve consensus.<br>Study authors will be contacted to request further details if study infromation is insufficient.<br>We will present a 'Risk of bias summary' figure and 'Risk of bias graph' .
Data synthesis	15a	7	An overall estimate of the intervention effect will be determined by combining more than one study with meta-analysis when the same intervention and outcomes with comparable methods in a similar population are examined.
	15b	7-8	We plan to estimate the overall risk ratio (RR) using the Mantel-Haenszel method for dichotomous outcomes. For continuous outcomes, the MD or SMD will be estimated based on the same or different scales for measuring the same outcome.<br>If statistical heterogeneity is low, we will perform a meta-analysis with fixed-effect models. Otherwise, random-effect models will be used to provide a more conservative estimate of the difference.
	15c	8	We will perform subgroup analysis based on the type of intervention, if possible. All subgroup analysis results will be presented in forest plots.<br>When more than 80% of participants come from one or two trials of included studies, the fixed-effect method could provide a more conservative estimate of the difference. These studies will be analysed as one of the subgroups.<br>If the heterogeneity is statistically significant ($P<0.10$), we will conduct meta-regression analysis to investigate the heterogeneity.<br>We will perform sensitivity analyses to determine whether our findings are robust.

For peer review only

			We plan to conduct sensitivity analysis for the following factors:<br>● Impact of model chosen: fixed-effect model versus random-effect model<br>● Risk of bias of included studies: excluding studies with high or unclear risk of bias<br>● Impact of trial design: excluding studies with crossover design<br>● Impact of the missing data: excluding studies which lost over 50% of patients to follow-up
	15d	8	We will provide a narrative summary of results from individual studies when it is impossible to conduct a meta-analysis.
Meta-bias(es)	16	7	If at least 10 eligible studies are included in a meta-analysis, publication bias will be explored by assessing funnel plot for signs of asymmetry. If asymmetry is identified, possible reasons will be explored.
Confidence in cumulative evidence	17	8	Two review authors (MP and JZ) will independently assess the quality of evidence for each outcome using the GRADE approach. Five factors (study limitations, imprecision, inconsistency, indirectness, and publication bias) could decrease the quality of evidence. The quality of evidence will fall into one of four categories from high to very low.<br>We will create 'summary of findings' tables for all outcomes using GRADEpro GDT. They will include the number of studies, quality assessment, summary of findings, and importance of the outcome.

For peer review only

BMJ Open

Probiotics, prebiotics and synbiotics for chronic kidney disease: protocol for a systematic review and meta-analysis

Journal:	*BMJ Open*
Manuscript ID	bmjopen-2017-020863.R2
Article Type:	Protocol
Date Submitted by the Author:	07-May-2018
Complete List of Authors:	Pei, Ming; First Teaching Hospital of Tianjin University of Traditional Chinese Medicine, Department of Nephrology<br>Wei, Lijuan; Tianjin University of Traditional Chinese Medicine, Graduate College<br>Hu, Shouci; Tianjin University of Traditional Chinese Medicine, Graduate College<br>Yang, Bo; First Teaching Hospital of Tianjin University of Traditional Chinese Medicine, Department of Nephrology<br>Si, Jinhua; Tianjin University of Traditional Chinese Medicine, Library<br>Yang, Hongtao; First Teaching Hospital of Tianjin University of Traditional Chinese Medicine, Department of Nephrology<br>Zhai, Jingbo; Tianjin University of Traditional Chinese Medicine, Research institute of Traditional Chinese Medicine
**Primary Subject Heading**:	Renal medicine
Secondary Subject Heading:	Renal medicine
Keywords:	Probiotics, prebiotics, synbiotics, chronic kidney disease, systematic review, meta-analysis

SCHOLARONE™
Manuscripts

Probiotics, prebiotics and synbiotics for chronic kidney disease: protocol for a systematic review and meta-analysis

Ming Pei[1][1], Lijuan Wei[2], Shouci Hu[2], Bo Yang[1], Jinhua Si[3], Hongtao Yang[1], Jingbo Zhai[4]

[1] Department of Nephrology, First Teaching Hospital of Tianjin University of Traditional Chinese Medicine, 314 Anshanxi Road, Nankai District, Tianjin 300193, China;

[2] Graduate College, Tianjin University of Traditional Chinese Medicine, 312 Anshanxi Road, Nankai District, Tianjin 300193, China;

[3] Library, Tianjin University of Traditional Chinese Medicine, 312 Anshanxi Road, Nankai District, Tianjin 300193, China;

[4] Research Institute of Traditional Chinese Medicine, Tianjin University of Traditional Chinese Medicine, 312 Anshanxi Road, Nankai District, Tianjin 300193, China;

ABSTRACT

Introduction:

There is a growing interest in probiotic, prebiotic and synbiotic supplements for patients with chronic kidney disease (CKD). However, a systematic review and evaluation is lacking. The purpose of the present study is to assess the efficacy and safety of probiotics, prebiotics and synbiotics for non-dialysis or non-renal transplant patients with CKD.

Methods and analysis:

An extensive literature search will be undertaken to identify potentially eligible studies from electronic databases including PubMed (1946 to present), EMBASE (1974 to present), Web of Science (1900 to present), and the Cochrane Central Register of Controlled Trials (CENTRAL, all years). No language restriction will be applied to the search. Both parallel and crossover randomised controlled trials will be included. The risk of bias of each included study will be assessed using the Cochrane Risk of Bias Tool. The primary outcome measures are uraemic toxins. Secondary outcomes include kidney function, adverse cardiovascular events, all-cause mortality, cause-specific death, progression to end-stage kidney disease, quality of life, gastrointestinal function and adverse events. Data will be synthesised using

[1] First author: Ming Pei (tjtcmpeiming@126.com) as show in ScholarOne. Ming Pei and Lijuan Wei (tcmweilijuan@163.com) contributed equally to this paper.

Corresponding author: Hongtao Yang (tjtcmht@126.com) as show in ScholarOne. Hongtao Yang (tjtcmht@126.com) and Jingbo Zhai (zhaijingbo@foxmail.com) contributed equally to this paper.

For peer review only - http://bmjopen.bmj.com/site/about/guidelines.xhtml

appropriate statistical methods. The quality of evidence for each outcome will be assessed using the GRADE approach.

Ethics and dissemination:

No ethical approval is required as no primary data will be collected. We will publish findings from this systematic review in a peer-reviewed scientific journal, and the data set will be made freely available.

This systematic review protocol has been registered in the International Prospective Register for Systematic Reviews (PROSPERO: CRD42017079177).

Strengths and limitations of this study

- This is the first systematic review to investigate the efficacy and safety of probiotics, prebiotics or synbiotics for non-dialysis or non-renal transplant patients with chronic kidney disease (CKD).
- The search strategy was developed by a medical librarian with ten years' experience as an information specialist.
- This protocol was developed following the Preferred Reporting Items for Systematic Review and Meta-Analysis Protocols (PRISMA-P) 2015 statement.
- Study heterogeneity is likely to pose challenges for this meta-analysis.

BACKGROUND

Chronic kidney disease (CKD) is one of the most common chronic diseases worldwide[1]. According to the KDIGO (Kidney Disease: Improving Global Outcomes) Guideline 2012[2], CKD is defined as abnormalities of kidney structure or function, present for more than 3 months, with implications for health, and is categorised into five stages according to severity using estimated glomerular filtration rate (GFR) or into three stages based on albuminuria[3].

The incidence of CKD is subject to variation worldwide[4]. The global prevalence of CKD is estimated to vary between 8 and 16%, and the number is 10.8% in China and 13.1% in the US[5,6]. Diabetes and hypertension are the main risk factors for CKD[4]. The global prevalence of CKD is likely to increase in view of the growing number of patients with diabetes and/or hypertension.

Under normal conditions homeostasis is maintained owing to interactions between the host and intestinal microflora[7]. However, quantitative and qualitative alterations in the intestinal microflora are present in CKD patients[7]. This dysbiotic intestinal microflora is characterised by an increase in the pathogenic flora relative to the symbiotic flora[7]. The pathogenic gut microbiota produce uraemic toxins, in particular indoxyl sulphate (IS) and p-cresyl sulphate (PCS) among others, which have been associated with increased inflammation, greater oxidative stress[7,8], and

For peer review only - http://bmjopen.bmj.com/site/about/guidelines.xhtml

higher risk for cardiovascular disease (CVD), progression of CKD and death due to CKD[9,10].

Probiotics refer to the living microorganisms which colonise or implant in the host's gastrointestinal environment and exert beneficial health effects[11,12]. Prebiotics are defined as non-digestible food ingredients that induce the growth and/or activity of beneficial microorganisms in the host[11,12]. Synbiotics are a mixture of probiotics and prebiotics.

Recently, probiotics, prebiotics or synbiotics have been reported to reduce inflammation, improve kidney function and retard progression of CKD by restoring the symbiosis of gut microflora in CKD patients. A randomised trial found synbiotics decreased serum PCS without reducing serum IS in non-dialysis CKD[13]. A pilot study suggested probiotic dietary supplements are more effective than placebo in reducing blood urea nitrogen (BUN) and improving the quality of life of patients with stage 3 or 4 CKD[14]. Another study found synbiotics delayed CKD progression[9].

Guldris et al. performed a narrative review of gut microbiota in relation to chronic kidney disease in 2017[7]. Their findings are open to dispute due to evident methodological flaws. First, literature retrieval was insufficient, largely because a comprehensive search strategy was not established. Second, the quality of the included studies was not assessed against a validated tool. Third, no statistical analysis was done and the evidence was left ungraded.

Another systematic review found prebiotic and probiotic therapies reduced IS and PCS in patients with end stage kidney disease (ESKD) on haemodialysis[15]. However, it is unclear whether the results hold true for other CKD patients. To our knowledge, previous systematic reviews have not fully addressed the research topic in question.

The objective of this study is to provide a comprehensive systematic review of the available evidence on the efficacy and safety of probiotics, prebiotics and synbiotics for the management of non-dialysis or non-renal transplant patients with CKD.

METHODS:

This protocol was developed following the Preferred Reporting Items for Systematic reviews and Meta-Analyses guidelines for protocols (PRISMA-P) [16].

Types of studies

We will include parallel and crossover randomised controlled trials (RCTs). Quasi-RCTs, controlled clinical trials (CCTs), controlled before and after trials, and cluster-RCTs will be excluded to minimise biased estimates of treatment effects[12].

Types of participants

Non-dialysis or non-renal transplant patients at any stage of CKD (as defined by

For peer review only - http://bmjopen.bmj.com/site/about/guidelines.xhtml

KDIGO) will be included[17]. We will consider patients of any age, both sexes, any ethnicity, and studies in any clinical setting. Trials including healthy people or non-CKD patients in the control group will be excluded.

Types of interventions

Experimental interventions

Probiotics, prebiotics and synbiotics regardless of dose, frequency, duration or route of delivery, and in combination or as the only preparation will be included[18].

Comparator interventions

Placebo, no treatment, or active pharmacological or non-pharmacological treatment (regardless of dose, frequency, duration, route of delivery or setting, and in combination or as the only preparation) will be included. We will exclude RCTs with probiotics, prebiotics and synbiotics in both arms.

Types of outcome measures:

Primary outcome

The primary outcome measures are uraemic toxins (including but not limited to phenols and indoles). Phenols include p-cresol, p-cresyl sulphate (PCS) and p-cresyl glucuronide, et al[7]. Indoles include indoxyl sulfate (IS) and indoleacetic acid, et al[7].

Secondary outcomes

The secondary outcome measures are listed below:

- Kidney function measures (including but not limited to blood urea nitrogen, glomerular filtration rate, creatinine clearance and serum creatinine);
- Major adverse cardiovascular events as defined by the investigator (including but not limited to myocardial infarction, coronary artery disease, heart failure, cerebrovascular disease and peripheral vascular disease);
- All-cause mortality and cause-specific death, such as cardiovascular mortality, sudden death and infection-related mortality, etc;
- Progression to ESKD requiring renal replacement therapy (RRT: haemodialysis, peritoneal dialysis or kidney transplantation);
- Quality of life measured by a validated scale, such as the Kidney Disease Quality of Life (KDQoL);
- Gastrointestinal (GI) function (including but not limited to improvement in GI symptoms, transit time and tolerance);
- Adverse events.

Search methods for identification of studies

Electronic searches

For peer review only - http://bmjopen.bmj.com/site/about/guidelines.xhtml

An extensive literature search will be undertaken to identify potentially eligible studies in electronic databases including PubMed (1946 to present), EMBASE (1974 to present), Web of Science (1900 to present) and the Cochrane Central Register of Controlled Trials (CENTRAL, all years).

The search strategy was developed by a medical librarian (JS) with ten years' experience as an information specialist, taking into consideration of the search terms used in previous reviews[17,18]. The search strategy for the PubMed database is provided in Appendix 1. The search terms are adapted properly to cater to each database. No language restriction will be applied to the search.

Searching other resources

We will search ClinicalTrials.gov and the World Health Organization International Clinical Trials Registry Platform (ICTRP) for relevant unpublished or ongoing studies. Google Scholar will be searched using key words to identify grey literature[19]. The reference lists of all retrieved studies and previous systematic reviews will be checked.

Data collection and analysis

Selection of studies

Two review authors (LW and BY) will independently run all the literature searches. The identified references from electronic database searching and other resources will be imported into the EndNote software[20]. Duplicate records will be removed. The title and abstract of each citation will be screened to identify potentially eligible studies, followed by full-text review to confirm inclusion. Any disagreement will be resolved by consensus or consultation with a third reviewer (JZ).

Including duplicated data in a meta-analysis may lead to overestimation of the intervention effects[21]. If uncertainties remain in justifying multiple publications from a single data set, the author of the original reports will be contacted for clarification[21].

A PRISMA flow diagram will be provided to state the process of study selection.

Data extraction and management

Two review authors (SH and HY) will independently extract data from included studies using a standardised data extraction form. The form will be specially designed and piloted by the responsible reviewer. Any disagreement will be resolved through discussion. A third reviewer (JZ) will be consulted for a final decision if consensus can not be reached.

- Study characteristics: title, author, publication year, design, sample size, funding source, and use of randomisation, allocation concealment, blinding and control.

For peer review only - http://bmjopen.bmj.com/site/about/guidelines.xhtml

- Participant characteristics: age, sex, number in each group and CKD diagnostic criteria.
- Intervention details: intervention treatment, comparator treatment, dose, route of administration and number of cases included in statistical analysis.
- Outcome measures reported regarding efficacy and safety.

Assessment of risk of bias for included studies

Two review authors (MP and JS) will independently assess the risk of bias for each included study using the Cochrane Risk of Bias Tool in RevMan 5.3[21].

The methodological quality of included studies will be assessed from seven aspects, including random sequence generation, allocation concealment, blinding of participants and personnel, blinding of outcome assessment, incomplete outcome data, selective reporting and other potential sources of bias[21].

The reviewers will evaluate the seven risk of bias items one by one and grade the risk for each item as either high, low or unclear. Disagreements will be resolved through discussion. A third reviewer (JZ) will be consulted to achieve consensus whenever necessary.

The authors of the original study will be contacted for further details if the information reported is insufficient to make a judgement.

Results of the assessment will be presented in a risk of bias summary figure and a risk of bias graph[21].

Measures of treatment effect

The risk ratio (RR) with its 95% confidence intervals (CIs) will be calculated for dichotomous outcomes.

The mean difference (MD) or standardised mean difference (SMD) with 95% CIs will be calculated for continuous outcomes.

The MD will be used if the same scale is used across different studies to measure an outcome. The SMD will be employed if different scales are used to measure the same outcome.

If a mixture of endpoint data and change from baseline data are reported for continuous outcomes in the included studies, subgroup analysis will be performed separately and the effect size estimates of different subgroups will not be pulled[22].

A narrative description of the results will be provided if less than two trials are included for one outcome measure[22].

Unit of analysis issues

Only the data from the first phase of a crossover study will be analysed in this study [23].

For studies dealing with multiple treatment groups the review authors will make

For peer review only - http://bmjopen.bmj.com/site/about/guidelines.xhtml

multiple pair-wise comparisons between pairs of groups of interest to the review question[21], and take care to avoid repeated inclusion of the same study group.

Dealing with missing data

Data missing may occur for any outcome of interest. If necessary, we will contact authors of the original report for additional data. We will not fill missing data for any outcome in the primary analysis, but the impact of data missing will be assessed in the sensitivity analysis.

If the standard deviation (SD) for an outcome is not reported, we will calculate the SD according to data reported or additional data collected from the authors.

Any potential impact of missing data will be discussed in the 'Discussion' section of this review.

Assessment of heterogeneity

Statistical heterogeneity will be evaluated by visual inspection of the forest plot and the Chi-squared test. In addition, the I^2 statistical value will be calculated to quantify heterogeneity. Heterogeneity will be categorised according to the following rules.

- I^2 of 0%-40% as might not be important;
- I^2 of 30%-60% as may represent moderate heterogeneity;
- I^2 of 50%-90% as may represent substantial heterogeneity;
- I^2 of 75%-100% as considerable heterogeneity.

If the P-value from a Chi-squared test is less than 0.10 or $I^2 > 50\%$, suggesting detectable between-study variation[21,24], subgroup analysis considering pre-specified factors will be performed in search of possible explanation for statistical heterogeneity.

Assessment of reporting bias

Publication bias will be examined by assessing a funnel plot for signs of asymmetry if more than ten studies are included in a meta-analysis[23]. If asymmetry is identified, possible explanations will be attempted.

Data synthesis

An overall estimate of the intervention effect will be calculated by combining multiple study results in a meta-analysis on the condition that the same outcome of the same intervention was measured with comparable methods in a homogeneous population across studies.

The overall risk ratio (RR) for dichotomous data will be estimated using the Mantel-Haenszel method[23]. For continuous data the MD or SMD will be calculated

For peer review only - http://bmjopen.bmj.com/site/about/guidelines.xhtml

in different situations.

When statistical heterogeneity is low the fixed-effect model will be adopted. Otherwise, the random-effect model will be used to provide a more conservative estimate of the difference.

A descriptive summary of results from individual studies will be provided when it is impossible to conduct a meta-analysis.

Subgroup analysis and investigation of heterogeneity

Subgroup analysis will be conducted for different pair of comparisons. The results of subgroup analysis will be presented in forest plots.

If one or two trials contributed to more than 80% of participants in a meta-analysis, the fixed-effect method will be preferred to provide a more conservative estimation[25]. Also, these studies will be analysed in one subgroup[25].

A meta-regression analysis will be performed to investigate possible sources if heterogeneity is statistically significant ($P<0.10$).

Sensitivity analysis

We will perform sensitivity analysis to test the robustness of our findings taking into consideration the possible impact of the following factors:

- The model chosen for data pulling: to switch between fixed-effect model and random-effect model
- Methodological quality of included studies: studies with high or unclear risk of bias will be excluded
- Study design: studies with a crossover design will be excluded
- Missing data: studies with over half of patients lost to follow-up will be excluded

Summary of findings table

Two review authors (MP and JZ) will independently assess the quality of evidence for each outcome using the GRADE approach. Five factors (study limitations, imprecision, inconsistency, indirectness, and publication bias) could decrease the quality of evidence. The quality of evidence will fall into one of four categories from very low to high[26].

A Summary of Findings table will be created including all the outcome measures using the GRADEpro GDT[27].

Patient and Public Involvement

Patients were not involved in setting the research question or outcome measures, or in developing plans for the design or implementation of the study. There are no

For peer review only - http://bmjopen.bmj.com/site/about/guidelines.xhtml

plans to disseminate the results of the research to study participants or any relevant patient community.

AMENDMENTS

We will provide the date, description, and rationale of any modification in the event of protocol amendments.

DISSEMINATION

This systematic review will provide a comprehensive assessment of the efficacy and safety of probiotics, prebiotics or synbiotics for non-dialysis or non-renal transplant patients with CKD. The findings will be important for generating reliable recommendations for the clinical management of CKD.

We plan to publish systematic review findings in a peer-reviewed scientific journal and make the data sets openly accessible.

Protocol registration: This systematic review protocol has been registered in the International Prospective Register for Systematic Reviews (PROSPERO-CRD42017079177).

Contributors:

JZ, MP and HY conceived the study. HY provided general guidance for drafting the protocol. JZ and MP drafted the protocol. JS designed the search strategy. MP, LW, SH, BY, JS, HY and JZ drafted the manuscript. MP, LW, SH, BY, JS, HY and JZ reviewed and revised the manuscript for intellectual content. All authors have read and approved the final version of the manuscript.

Acknowledgements:

The authors thank Wei Mu for revising the English language of the manuscript.

Funding:

This study is supported by the National Natural Science Foundation of China [Grant number 81703860], the National Natural Science Foundation of China [rant number 81673909] and Tianjin Science and Technology Program: Tianjin TCM Clinical Medicine Research Center [No. 15ZXLCSY00020].

Competing interests: None declared.

Ethics approval: Not required.

For peer review only

Provenance and peer review: Not commissioned; externally peer reviewed.

Data sharing statement: Findings from this systematic review will be published in a peer-reviewed scientific journal, and the data sets will be made freely available.

Open access: This is an Open Access article distributed in accordance with the terms of the Creative Commons Attribution (CC BY 4.0) license, which permits others to distribute, remix, adapt and build upon this work, for commercial use, provided the original work is properly cited. See: http://creativecommons.org/licenses/by/4.0/

References:

[1] Luis Vitetta, Glenda Gobe. Uremia and chronic kidney disease: The role of the gutmicroflora and therapies with pro- and prebiotics. Mol Nutr Food Res 2013;57(5):824-32.

[2] Kidney Disease: Improving Global Outcomes (KDIGO) CKD Work Group. KDIGO clinical practice guideline for the evaluation and management of chronic kidney disease. Kidney Int Suppl. 2013;3:1-150.

[3] Paul E. Stevens, Adeera Levin, Kidney Disease: Improving Global Outcomes Chronic Kidney Disease Guideline Development Work Group Members. Evaluation and Management of Chronic Kidney Disease: Synopsis of the Kidney Disease: Improving Global Outcomes 2012 Clinical Practice Guideline. Ann Intern Med 2013;158(11):825-30.

[4] Angela C Webster, Evi V Nagler, Rachael L Morton, et al. Chronic kidney disease. Lancet 2017;389:1238-52.

[5] Vivekanand Jha, Guillermo Garcia-Garcia, Kunitoshi Iseki, et al. Chronic kidney disease: global dimension and perspectives. Lancet 2013;382(9888):260-72.

[6] Csaba P Kovesdy, Kamyar Kalantar-Zadeh. Enter the dragon: a Chinese epidemic of chronic kidney disease? Lancet 2012;379(9818):783-5.

[7] Secundino Cigarran Guldris, Emilio González Parra, Aleix Cases Amenós. Gut microbiota in chronic kidney disease. Mefrologia, 2017,37(1):9-19.

[8] Megan Rossi, David W. Johnson, Mark Morrison, et al. SYNbiotics Easing Renal failure by improving GutmicrobiologY (SYNERGY): a protocol of placebo-controlled randomised cross-over trial. BMC Nephrology 2014;15:106.

[9] B. Guida, R. Germanò, R. Trio, et al. Effect of short-term synbiotic treatment on plasma p-cresol levels in patients with chronic renal failure: A randomized clinical trial. Nutr Metab Cardiovasc Dis 2014;24(9):1043-9.

[10] Cheng-Jui Lin, Vincent Wu, Pei-Chen Wu, et al. Meta-Analysis of the Associations of p-Cresyl Sulfate (PCS) and Indoxyl Sulfate (IS) with Cardiovascular Events and All-Cause Mortality in Patients with Chronic Renal Failure. Plos one

For peer review only - http://bmjopen.bmj.com/site/about/guidelines.xhtml

2015;10(7):e0132589.

[11] Jürgen Schrezenmeir and Michael de Vrese. Probiotics, prebiotics, and synbiotics- approaching a definition. Am J Clin Nutr 2001;73(2 Suppl): 361S-364S.

[12] Lulong bo, jinbao li, tianzhu tao, et al. Probiotics for preventing ventilator-associated pneumonia. Cochrane Database Syst Rev 2014;10:CD009066.

[13] Megan Rossi, David W. Johnson, Mark Morrison, et al. Synbiotics Easing Renal Failure by Improving Gut Microbiology (SYNERGY): A Randomised Trial. Clin J Am Soc Nephrol 2016;11(2):223-31.

[14] Ranganathan N, Ranganathan P, Friedman EA, et al. Pilot Study of Probiotic Dietary Supplementation for Promoting Healthy Kidney Function in Patients with Chronic Kidney Disease. Adv Ther 2010;27(9):634-647.

[15] Megan Rossi, Kerenaftali Klein, David W. Johnson, et al. Pre-, Pro-, and Synbiotics: Do They Have a Role in Reducing Uremic Toxins? A Systematic Review and Meta-Analysis. Int J Nepphrol 2012;2012:673631.

[16] Larissa Shamseer, David Moher, Mike Clarke, et al. Preferred reporting items for systematic review and meta-analysis protocols (PRISMA-P) 2015: elaboration and explanation. BMJ 2015;350:g7647.

[17] Natale P, Ruospo M, Saglimbene VM, et al. Interventions for improving sleep quality in people with chronic kidney disease. Cochrane Database Syst Rev 2017;4:CD012625.

[18] Chowdhurg A, Varadhan K, Neal K, et al. Perioperative prebiotics, probiotics or synbiotics for elective abdominal surgery in adults. Cochrane Database Syst Rev 2011;7:CD009246.

[19] Theodosia Adom, Thandi Puoane, Anniza De Villiers, et al. Protocol for systematic review of school-based interventions to prevent and control obesity in African learners. BMJ Open 2017;7(3):e013540.

[20] Lorainne Tudor Car, Rifat Atun. eLearning for health system leadership and management capacity building: a protocol for a systematic review. BMJ Open 2017;7(8):e017050.

[21] Higgins JPT, Green S. Cochrane Handbook for Systematic Reviews of Interventions Version 5.1.0 (updated March 2011). The Cochrane Collaboration, 2011. http://www.handbook.cochrane.org.

[22] Bae S, Kamynina E, Farinola AF, et al. Provision of folic acid for reducing arsenic toxicity in arsenic-exposed children and adults. Cochrane Database Syst Rev 2017:5:CD012649.

[23] Aamann L, Dam G, Rinnov A, et al. Physical exercise for people with cirrhosis. Cochrane Database Syst Rev 2017:6:CD012678.

[24] Susanne Roehr, Alexander Pabst, Tobias Luck, et al. Secular trends in the incidence of dementia in high-income countries: a protocol of a systematic review and

For peer review only - http://bmjopen.bmj.com/site/about/guidelines.xhtml

a planned meta-analysis. BMJ Open 2017;7(4):e013630.

[25] Browning GG, Howell P, Whitmer WM, et al. Unilateral versus bilateral hearing aids for bilateral hearing impairment in adults. Cochrane Database Syst Rev 2017:5:CD012665.

[26] Gordon Guyatt, Andrew D. Oxman, Elie Akl, et al. GRADE guidelines 1. Introduction-GRADE evidence profiles and summary of findings tables. J Clin Epidemiol 2011;64(4):383-394.

[27] GRADEpro GDT. GRADEpro Guideline Development Tool. 2015. https://gradepro.org.

For peer review only

For peer review only - http://bmjopen.bmj.com/site/about/guidelines.xhtml

Appendix 1.

The search strategy for the Pubmed database:

1. Kidney Diseases/
2. Renal Insufficiency/
3. exp Renal Insufficiency, Chronic/
4. (kidney disease* or renal disease* or kidney failure or renal failure or kidney insufficienc* or renal insufficienc* or frasier syndrome).tw.
5. (ESRF or ESKF or ESRD or ESKD).tw.
6. (CKF or CKD or CRF or CRD).tw.
7. or/1-6
8. Probiotics/
9. probiotic*.tw.
10. Synbiotics/
11. synbiotic*.tw.
12. Prebiotics/
13. prebiotic*.tw.
14. exp Lactobacillus/
15. Lactobacillus.tw.
16. lactobacilli*.tw.
17. exp Bifidobacterium/
18. bifidobacteri*.tw.
19. exp Bacillus/
20. bacilli*.tw.
21. bacillu*.tw.
22. Clostridium butyricum/
23. clostridium butyricum.tw.
24. Streptococcus thermophilus/
25. streptococcus thermophill*.tw.
26. exp Escherichia coli/
27. (escherichia coli or E coli or EAggEC).tw.

For peer review only

28. exp Propionibacterium/
29. propionibacteria*.tw.
30. propionibacterium freudenreich*.tw.
31. exp Enterococcus faecium/
32. enterococcus faecium.tw.
33. streptococcus faecium.tw.
34. exp Saccharomyces/
35. (saccharomyces boulardi or Saccharomyces cerevisiae or Baker Yeast* or S cerevisiae or Bakers Yeast or Brewer* Yeast).tw.
36. vsl3.tw.
37. vsl 3.tw.
38. ganedenBc.tw.
39. lafti b94.tw.
40. mutaflor.tw.
41. actimel.tw.
42. cultura.tw.
43. yakult.tw.
44. l casei.tw.
45. shirota.tw.
46. Enterobacteriaceae/
47. proviva.tw.
48. vifit.tw.
49. verum.tw.
50. bio K.tw.
51. multibionta.tw.
52. oligofructose.mp
53. Inulin/
54. Inulin.tw
55. or/8-54
56. 7 and 55

For peer review only

57. randomized controlled trial.pt.
58. controlled clinical trial.pt.
59. randomised.tw.
60. randomized.tw.
61. placebo.tw.
62. randomly.tw.
63. trial.tw.
64. groups.tw.
65. or/57-64
66. exp animals/ not humans.sh.
67. 65 not 66
68. 56 and 67

For peer review only

PRISMA-P 2015 checklist

Section and topic	Item No	Page	Checklist item
Administrative information			
Title			
Identification	1a	1	Probiotics, prebiotics and synbiotics for chronic kidney disease: protocol for a systematic review and meta-analysis
Update	1b		No
Registration	2	2	This protocol has been registered on PROSPERO (CRD42017079177).
Authors:			
Contact	3a	1	Ming Pei[1], Lijuan Wei[2], Shouci Hu[2], Bo Yang[1], Jinhua Si[3], Hongtao Yang[1*], Jingbo Zhai[4*]<br>[1] Department of Nephrology, First Teaching Hospital of Tianjin University of Traditional Chinese Medicine, 314 Anshanxi Road, Nankai District, Tianjin 300193, China; [2] Graduate College, Tianjin University of Traditional Chinese Medicine, 312 Anshanxi Road, Nankai District, Tianjin 300193, China; [3]Library of Tianjin University of Traditional Chinese Medicine, 312 Anshanxi Road, Nankai District, Tianjin 300193, China; [4] Research institute of Traditional Chinese Medicine, Tianjin University of Traditional Chinese Medicine, 312 Anshanxi Road, Nankai District, Tianjin 300193, China.<br>Ming Pei and Lijuan Wei contributed equally.<br>Correspondence to Hongtao Yang, tjtcmht@126.com; Jingbo Zhai, zhaijingbo@foxmail.com
Contributions	3b	9	JZ, MP and HY conceived the study. HY provided general guidance to the drafting of the protocol. JZ and MP drafted the protocol. JS designed the search strategy. MP, LW, SH, BY, JS, HY and JZ drafted the manuscript. MP, LW, SH, BY, JS, HY and JZ reviewed and revised the manuscript.

For peer review only

			All authors have read and approved the final version of the manuscript.
Amendments	4	9	We will provide the date, description, and rationale of any modification in the event of protocol amendments.
Support:			
Sources	5a	9	This study is supported by the National Natural Science Foundation of China [Grant number 81703860], the National Natural Science Foundation of China [rant number 81673909] and Tianjin Science and Technology Program: Tianjin TCM Clinical Medicine Research Center [No. 15ZXLCSY00020].
Sponsor	5b	9	JZ, MP and HY are sponsors.
Role of sponsor or funder	5c	9	MP and HY conceived the study. HY provided general guidance to the drafting of the protocol. JZ and MP drafted the protocol. MP, HY and JZ drafted the manuscript. MP, HY and JZ reviewed and revised the manuscript.
Introduction			
Rationale	6	2-3	There is a growing interest in probiotics, prebiotics and synbiotics supplements for patients with chronic kidney disease (CKD). However, no comprehensive systematic review has been conducted to evaluate the efficacy and safety of probiotics, prebiotics or sybiotics for CKD patients without dialysis and renal transplantation.
Objectives	7	3	The objective of this study is to provide a comprehensive systematic review of the available evidence on the efficacy and safety of probiotics, prebiotics and synbiotics for the management of non-dialysis or non-renal transplant patients with CKD.
Methods			
Eligibility criteria	8	3-4	Types of studies<br>We will include parallel and crossover randomised controlled trials (RCTs). Quasi-RCTs,

For peer review only

			controlled clinical trials (CCTs), controlled before and after trials, and cluster-RCTs will be excluded to minimise biased estimates of treatment effects[12].<br>Types of participants<br>Non-dialysis or non-renal transplant patients at any stage of CKD (as defined by KDIGO) will be included[17]. We will consider patients of any age, both sexes, any ethnicity, and studies in any clinical setting. Trials including healthy people or non-CKD patients in the control group will be excluded.<br>Types of interventions<br>Experimental interventions<br>Probiotics, prebiotics and synbiotics regardless of dose, frequency, duration or route of delivery, and in combination or as the only preparation will be included[18].<br>Comparator interventions<br>Placebo, no treatment, or active pharmacological or non-pharmacological treatment (regardless of dose, frequency, duration, route of delivery or setting, and in combination or as the only preparation) will be included. We will exclude RCTs with probiotics, prebiotics and synbiotics in both arms.<br>Types of outcome measures:<br>Primary outcome<br>The primary outcome measures are uraemic toxins (including but not limited to phenols and indoles). Phenols include p-cresol, p-cresyl sulphate (PCS) and p-cresyl glucuronide, et al[7]. Indoles include indoxyl sulfate (IS) and indoleacetic acid, et al[7].<br>Secondary outcomes<br>The secondary outcome measures are listed below:<br>● Kidney function measures (including but not limited to blood urea nitrogen, glomerular filtration

For peer review only

			rate, creatinine clearance and serum creatinine);<br>● Major adverse cardiovascular events as defined by the investigator (including but not limited to myocardial infarction, coronary artery disease, heart failure, cerebrovascular disease and peripheral vascular disease);<br>● All-cause mortality and cause-specific death, such as cardiovascular mortality, sudden death and infection-related mortality, etc;<br>● Progression to ESKD requiring renal replacement therapy (RRT: haemodialysis, peritoneal dialysis or kidney transplantation);<br>● Quality of life measured by a validated scale, such as the Kidney Disease Quality of Life (KDQoL);<br>● Gastrointestinal (GI) function (including but not limited to improvement in GI symptoms, transit time and tolerance);<br>● Adverse events.
Information sources	9	5	An extensive literature search will be undertaken to identify potentially eligible studies in electronic databases including PubMed (1946 to present), EMBASE (1974 to present), Web of Science (1900 to present) and the Cochrane Central Register of Controlled Trials (CENTRAL, all years). We will search ClinicalTrials.gov and World Health Organization International Clinical Trials Registry Platform (ICTRP) to identify unpublished or ongoing studies. Google Scholar will be searched using key words to identify grey literature.
Search strategy	10	5	An extensive literature search will be undertaken to identify potentially eligible studies in electronic databases including PubMed (1946 to present), EMBASE (1974 to present), Web of Science (1900 to present) and the Cochrane Central Register of Controlled Trials (CENTRAL, all years).

For peer review only

			The search strategy was developed by a medical librarian (JS) with ten years' experience as an information specialist, taking into consideration of the search terms used in previous reviews. The search strategy for the PubMed database is provided in Appendix 1. The search terms are adapted properly to cater to each database. No language restriction will be applied to the search.<br>We will search ClinicalTrials.gov and the World Health Organization International Clinical Trials Registry Platform (ICTRP) for relevant unpublished or ongoing studies. Google Scholar will be searched using key words to identify grey literature. The reference lists of all retrieved studies and previous systematic reviews will be checked.
Study records			
Data management	11a	5	The identified references from electronic database searching and other resources will be imported into the EndNote software. Duplicate records will be removed.
Selection process	11b	5	Two review authors (LW and BY) will independently run all the literature searches. The identified references from electronic database searching and other resources will be imported into the EndNote software. Duplicate records will be removed. The title and abstract of each citation will be screened to identify potentially eligible studies, followed by full-text review to confirm inclusion. Any disagreement will be resolved by consensus or consultation with a third reviewer (JZ).<br>Including duplicated data in a meta-analysis may lead to overestimation of the intervention effects. If uncertainties remain in justifying multiple publications from a single data set, the author of the original reports will be contacted for clarification.<br>A PRISMA flow diagram will be provided to state the process of study selection.
Data collection process	11c	5	Two review authors (SH and HY) will independently extract data from included studies using a standardised data extraction form. The form will be specially designed and piloted by the responsible reviewer. Any disagreement will be resolved through discussion. A third reviewer (JZ) will be

For peer review only

			consulted for a final decision if consensus can not be reached.
Data items	12	5-6	● Study characteristics: title, author, publication year, design, sample size, funding source, and use of randomisation, allocation concealment, blinding and control.<br>● Participant characteristics: age, sex, number in each group and CKD diagnostic criteria.<br>● Intervention details: intervention treatment, comparator treatment, dose, route of administration and number of cases included in statistical analysis.<br>● Outcome measures reported regarding efficacy and safety.
Outcomes and prioritization	13	4	Primary outcome<br>The primary outcome measures are uraemic toxins (including but not limited to phenols and indoles). Phenols include p-cresol, p-cresyl sulphate (PCS) and p-cresyl glucuronide, et al[7]. Indoles include indoxyl sulfate (IS) and indoleacetic acid, et al[7].<br>Secondary outcomes<br>The secondary outcome measures are listed below:<br>● Kidney function measures (including but not limited to blood urea nitrogen, glomerular filtration rate, creatinine clearance and serum creatinine);<br>● Major adverse cardiovascular events as defined by the investigator (including but not limited to myocardial infarction, coronary artery disease, heart failure, cerebrovascular disease and peripheral vascular disease);<br>● All-cause mortality and cause-specific death, such as cardiovascular mortality, sudden death and infection-related mortality, etc;<br>● Progression to ESKD requiring renal replacement therapy (RRT: haemodialysis, peritoneal dialysis or kidney transplantation);<br>● Quality of life measured by a validated scale, such as the Kidney Disease Quality of Life

For peer review only

			(KDQoL);<br>● Gastrointestinal (GI) function (including but not limited to improvement in GI symptoms, transit time and tolerance);<br>● Adverse events.
Risk of bias in individual studies	14	6	Two review authors (MP and JS) will independently assess the risk of bias for each included study using the Cochrane Risk of Bias Tool in RevMan 5.3.<br>The methodological quality of included studies will be assessed from seven aspects, including random sequence generation, allocation concealment, blinding of participants and personnel, blinding of outcome assessment, incomplete outcome data, selective reporting and other potential sources of bias.<br>The reviewers will evaluate the seven risk of bias items one by one and grade the risk for each item as either high, low or unclear. Disagreements will be resolved through discussion. A third reviewer (JZ) will be consulted to achieve consensus whenever necessary.<br>The authors of the original study will be contacted for further details if the information reported is insufficient to make a judgement.<br>Results of the assessment will be presented in a risk of bias summary figure and a risk of bias graph.
Data synthesis	15a	7	An overall estimate of the intervention effect will be calculated by combining multiple study results in a meta-analysis on the condition that the same outcome of the same intervention was measured with comparable methods in a homogeneous population across studies.
	15b	7-8	The overall risk ratio (RR) for dichotomous data will be estimated using the Mantel-Haenszel method. For continuous data the MD or SMD will be calculated in different situations.<br>When statistical heterogeneity is low the fixed-effect model will be adopted. Otherwise, the

For peer review only

			random-effect model will be used to provide a more conservative estimate of the difference.
	15c	8	Subgroup analysis and investigation of heterogeneity<br>Subgroup analysis will be conducted for different pair of comparisons. The results of subgroup analysis will be presented in forest plots.<br>If one or two trials contributed to more than 80% of participants in a meta-analysis, the fixed-effect method will be preferred to provide a more conservative estimation. Also, these studies will be analysed in one subgroup.<br>A meta-regression analysis will be performed to investigate possible sources if heterogeneity is statistically significant ($P<0.10$).<br>Sensitivity analysis<br>We will perform sensitivity analysis to test the robustness of our findings taking into consideration the possible impact of the following factors:<br>● The model chosen for data pulling: to switch between fixed-effect model and random-effect model<br>● Methodological quality of included studies: studies with high or unclear risk of bias will be excluded<br>● Study design: studies with a crossover design will be excluded<br>● Missing data: studies with over half of patients lost to follow-up will be excluded
	15d	8	A descriptive summary of results from individual studies will be provided when it is impossible to conduct a meta-analysis.
Meta-bias(es)	16	7	Publication bias will be examined by assessing a funnel plot for signs of asymmetry if more than ten studies are included in a meta-analysis. If asymmetry is identified, possible explanations will be attempted.

For peer review only

Confidence in cumulative evidence	17	8	Two review authors (MP and JZ) will independently assess the quality of evidence for each outcome using the GRADE approach. Five factors (study limitations, imprecision, inconsistency, indirectness, and publication bias) could decrease the quality of evidence. The quality of evidence will fall into one of four categories from very low to high.<br>A Summary of Findings table will be created including all the outcome measures using the GRADEpro GDT.

For peer review only
